# Unique insights into morphological characterization and functional adaptation of the scaly shank skin in aquatic and terrestrial birds

**DOI:** 10.1038/s41598-024-77650-w

**Published:** 2024-11-15

**Authors:** Fatma A. Madkour

**Affiliations:** Department of Anatomy and Embryology, Faculty of Veterinary Medicine, South Valley University, Qena, 83523 Egypt

**Keywords:** BBWT, EBD, Dermis, Epidermis, Largenhans cells, Scales, Telocytes, Cell biology, Systems biology, Zoology

## Abstract

The avian skin is a vital barrier against external effects and undergoes modification to adapt to the different ecosystems. The current study focused on the comprehensive study of the scaly shank skin of aquatic birds, Egyptian Balady Duck (EBD) as well as terrestrial birds, Broad Breasted White Turkey (BBWT) via gross anatomy, histology, and scanning electron microscopy with ED-XRF analysis. The shank skin color was yellow to black in EBD and creamy-white in BBWT. Gross anatomy exhibited two types of scales around the shank: scute and scutella scales in EBD and four types: scute, scutella, reticula, and cancella (interstitial) scales in BBWT. Most scales were non-overlapped and separated by sulci in both birds except those on the dorsum of the shank of BBWT. SEM of the descaled skin revealed an irregular surface due to keratinocytes defining a polygonal texture in EBD and Langerhans cells (a large oval-shaped cell body with abundant long projections) attached to neighboring keratinocytes in BBWT. Histologically, the epidermal and dermal layers varied among the different skin aspects of the shanks of both birds. Langerhans cells were seen within the basal cell layer of the stratum germinativum and collagen fibers of the dermal stratum superficiale. Melanocytes were observed in the stratum basale in EBD. In both birds, abundant telocytes and fibroblasts were distributed within the dermal layers, with excessive adipose tissue in the dermis of the shank skin of EBD. According to the results of the ED-XRF analysis of the scaly shank skin, elements and oxides were present in both species. In conclusion, the findings of the present study reflect the correlations between the functional morphology of shank skin and the bird habitats.

## Introduction

Recently, in ornithological literature, the part of the limb enclosing the tarsometatarsus between the ankle and toes has been known as the shank^1,2^. Even though, in previous decades, Coues^3^ reported that the tarsometatarsus is erroneously called the shank. Furthermore, Baumel, et al.^4^ mentioned that the shank or crus in mammals and birds lies between the knee and the ankle. The shank is an essential region in the avian body, but updated studies on its morphological features are uncommon. Therefore, a thorough study was conducted on the shank skin of two species of different habitats: aquatic birds, Egyptian Balady Duck (EBD) and terrestrial birds, Broad Breasted White Turkey (BBWT). Domestic ducks (*Anas boschas domesticus*) belong to the family Anatidae and are descended from mallards (Anas platyrhynchos). Domiati and balady ducks are the native breeds of duck^5^. However, domestic turkeys (*Meleagris gallopavo domesticus*) belong to the family Phasianidae and were brought by the Spanish from Central Mexico into Europe in the sixteenth century^6^, their breeding acquired several developments in the early twentieth century^7^. This species is a bird of worldwide economic importance^8^. BBWT is the most popular breed of domesticated turkey because its weight may exceed 30 kg^9^.

Each organism has its ecosystem in which it lives. To survive, all organisms need to adapt to their habitats. An adaptation is a modification in an organism’s body or behavior. The organism may adapt to its habitat in different ways. It may be a physical or structural adaptation, just as avian skin undergoes modification to produce specialized horny structures such as epidermal scales, feathers, beaks, and claws^10^. Birds are present all around the globe and in a wide variety of habitats, from deserts to aquatic habitats and from tropical jungles to Antarctic ice fields. Their ability to exist in different conditions is due primarily to their skin and feather. Weir and Lunam^11^ confirmed that the functional anatomy of avian skin is modified according to bird habitat. The histological morphology of the avian skin varies depending on the species, age, and integumentary region. The variations are likely responses to environmental stresses.

The avian body skin of the most modern species including the studied birds is covered with feathers except beaks, scales, digits, and claws^12^. While, many wild species (i.e., owls, eagles, ptarmigans, and grouses), some breeds of domestic chicken and pigeon, and earliest known birds (Archaeopteryx) exhibit foot feathering but not including their toes^13–17^.

The avian skin is similar to that of other higher vertebrates; but, except for the unprotected areas such as the lower legs, it is much thinner^10^. The feathered and featherless skin of the avian consists of the epidermis, dermis (corium), and subcutis (hypodermis)^12,13,18^. The avian epidermis is analogous to that of mammals^12^. It consists of 2 principal layers: the inner, living, stratum germinativum and the outer non-living, stratum corneum. The epidermal layers are thicker in the non-feathered regions^10,12,19^. The dermis is markedly different from that of mammals, consists of a stratum superficiale (superficial layer) and a stratum profundum (deep layer). The stratum profundum is divided into two sub layers: the stratum compactum and stratum laxum. Finally, an internal layer, the subcutis (hypodermis) consists of adipose tissue.

The non-feathered, scale-covered integument of the avian ankle, tarsometatarsus, and digits is known as podotheca. Generally, the medial and lateral surfaces of the tarsometatarsus are covered only by the scaly podotheca^4,12^. By contrast, the plantar and dorsal surfaces of the tarsometatarsus have bundles of flexor and extensor tendons interposed between the podotheca and bone^20^. The scaly covering (part of the podotheca) of the dorsal side of the tarsometatarsus is called Acrotarsium or acrometatarsium, and of digits is called the acropodium^4^.

Some recently published data reported that avian scales re-evolved after the evolution of feathers^18,21,22^. They originate in the embryo as flattened epidermal papillae overlying condensations of the dermis^10^. The avian scales are variable in size, shape, amount of overlap, and degree of fusion, even in areas of the same feet^4,13,23^. Interestingly, the avian scales can be categorized into 4 types: (1) Scute (Scutum) scales that are large, flat, polygonal, rounded, or conical raised areas, overlapped resemble those in reptiles, and located on the metatarsus (mainly anterior surface). (2) Scutella (Scutellum) scales that are small, not quite as large as scute and found on the caudal of the metatarsal region. (3) Reticulate scales that are radially symmetrical dome-shaped and located on the soles of the feet, homologous to reptilian tuberculate scales. (4) Cancella (interstitial) scales that are minute scales resemble the reticulate type but more variable in shape and located on the surface of the hock joint and on the lateral and medial surfaces of the tarsometatarsus^4,13,24,25^. Different types of avian scales are made up of both a-keratins and corneous beta-proteins (CBPs; formerly known as "b-keratins"), which are similar to the epidermal components of birds such as feathers, beaks, and claws. Reticulate scales are made up only of a-keratins^25,26^.

The present study included not only the skin of aquatic birds, Egyptian Balady Duck (*Anas boschas domesticus*) and terrestrial birds, White Broad Breasted Turkey (*Meleagris gallopavo domesticus*) but also its derivatives such as scales. This study is the first to shed light on the elemental analysis across the avian shank skin via ED-XRF to understand the role of the elemental composition in adaptation to environmental conditions. On the other hand, the elemental analysis provides new insights into the potential use of the shank skin as an animal protein that is a good source of mineral supplementation in the food industry. Furthermore, in future research, the elemental analysis via ED-XRF could stimulate the application of other biochemical analyses to extract collagen and gelatin from avian shank skin which might be employed in the biomedical and pharmaceutical industries.

## Materials and methods

### Bird specimen collection and preparation

This study was carried out on 30 healthy adult birds of both sexes: Egyptian Balady Ducks (EBD) (*Anas boschas domesticus*) (n = 15) and Broad Breasted White Turkeys (BBWT) (*Meleagris gallopavo domesticus*) (n = 15). Egyptian Balady Ducks were purchased from a village in Qena City, Egypt. Furthermore, Broad Breasted White Turkeys were collected from a local farm in Cairo governorate, Egypt. The average body weight of EBD and BBWT was 2.5 ± 1.5 kg and 16.63 ± 5.54 kg, respectively. The collection of the birds was performed in March 2022.

The birds were anesthetized with Anahal (isoflurane) solution and then sacrificed with a razor-sharp knife. These processes were carried out immediately upon the collection of the birds. Finally, the right and left legs of each bird were dissected from the body.

### Gross anatomy

For gross anatomy examination, the right and left legs of six birds of each species were washed, cleaned with distilled water and saline, and then fixed in 10% neutral buffered formalin (NBF). The mean length of the shank region was 30.76 ± 2.71 mm in EBD and 59.81 ± 1.34 mm in BBWT. The general morphology of the shank skin was investigated via a magnification lens. Moreover, the photographs were taken via a Samsung Phone camera (Samsung Galaxy A52s 5G/SM-A528BZWOMEA). The anatomical terminology in this study is based on Nomina Anatomica Avium^4^.

### Scanning *electron* microscopy

Shank skin samples of the dorsal, plantar, medial, and lateral areas (n = 3 birds of each species) were washed several times with phosphate buffer solution (ph = 7.3), fixed in a 4% glutaraldehyde solution, and then post-fixed in a 2% buffered osmium tetroxide solution^27^. The fixed samples were washed in 0.1 M cacodylate buffer and dehydrated in ascending grades of alcohol. Thereafter, the samples were exposed to the critical point drying process (Critical Point Drying Procedure Polaron E3000 CPD device)^28^ and coated with gold palladium. The samples were examined via a scanning electron microscope (JSM-5500 LV, JEOL Ltd. Japan) at the SVU Central Lab, Qena, Egypt. The photographs were taken via a camera attached at 20 kV for SEM (JSM-5500 LV) on different magnifications.

### Coloring of the scanning *electron* microscopy images

SEM images were colored using the Photo Filter 7.2.1 program to differentiate between various structures. The method was used by^29^.

### Histology

Shank skin samples of the dorsal, plantar, medial, and lateral aspects (n = 3 birds of each species) were cut with a sharp scalpel into small pieces (0.5 cm^3^) and then immersed in 10% neutral buffered formalin (NBF) for 24 h. The fixed samples were dehydrated in ascending grades of ethyl alcohol (70%, 80%, 90%, and 100%) and then cleared in xylene. Following embedding in paraffin wax, 4-5μm thick sections were taken via a Leica RM2235 microtome. The sections were stained with H&E and Masson’s trichrome^30^ as well as Picrosirius red stain (Polyscience Europ GmbH)^31^.

### Preparation of semithin sections

The samples were prepared for resin embedding^32^. They were fixed in 2.5% glutaraldehyde, 4% paraformaldehyde, and 1% osmium tetroxide. The samples were dehydrated and then embedded in Spur’s resin. Semithin sections (0.5um thickness) were stained with toluidine blue^33^. The prepared stained sections were analyzed and photographed via a light microscope (Leitz Dialux 20, Leica DMLS) and a digital camera (Canon Power shot A95).

### CMEIAS color segmentation

Improved computational technology and CMEIAS color segmentation were used to create negative images of some figures. This process has been done by the following steps: the image is opened with CMEIAS Color Segmentation, then choose “Process” from the menu items and subsequently click “Negative image”^34^. Several scholars^35,36^ have applied this process.

### Energy-dispersive X ray Fluorescence (ED-XRF) experimental procedure

ED-XRF spectrometry (ent AnalyzerElem JEOL JS 3222, Japan) in the Central Lab, SVU, Qena, Egypt, was used to analyze the elements of the scaly shank skin of both species. The sample powder was prepared by drying, grinding and then being transported to the laboratory. The following procedure was performed as previously documented by Madkour and Abdelsabour-Khalaf^37^. Briefly, the equipment was supported with a thermoelectrically Peltier cooled solid-state Si (Li) X-ray detector at a 50 kV and 50 W. The spectrometer was equipped with Edwards’s vacuum pump to investigate elements with lower atomic weights than titanium (Ti) and sodium (Na). An analog-to-digital converter and a pulse processor were used to collect the data. Three replicates of each measurement were performed and the average of the data was determined to ensure that the maximum accuracy was obtained. The acquired data of the elements and oxides concentrations are expressed in Tables 1 and 2.

**Table 1:** The elemental composition of the scaly shank skin of the Egyptian balady duck (EBD) and the broad breasted white turkey (BBWT).

Element	Bird		
	EBD		BBWT
	Line	Concentration	
Al (Aluminum)	Ka	10.887 Wt %	6.764 Wt %
Si (Silicone)	Ka	10.713 Wt %	11.336 Wt %
P (Phosphorus)	Ka	2.817 Wt %	1.887 Wt %
S (Sulphur)	Ka	16.252 Wt %	16.926 Wt %
Ti (Titanium)	Ka	3.445 Wt %	4.484 Wt %
Cr (Chromium)	Ka	0.220 Wt %	-
Mn (manganese)	Ka	0.315 Wt %	0.383 Wt %
Fe (Iron)	Ka	10.628 Wt %	8.714 Wt %
Ni (Nickel)	Ka	0.177 Wt %	1.127 Wt %
Cu (Copper)	Ka	0.199 Wt %	0.209 Wt %
Zn (Zinc)	Ka	0.843 Wt %	0.993 Wt %
Se (Selenium)	Ka	-	0.026 Wt %
Nb (Niobium)	Ka	-	0.142 Wt %
Zr (Zirconium)	Ka	0.075%	-
Ag (Silver)	La	6.558%	6.929 Wt %
Sn (Tin)	La	16.238%	23.548 Wt %
Sb (Antimony)	La	20.634%	16.533 Wt %

**Table 2:** Oxides concentration for the scaly shank skin of the Egyptian balady duck (EBD) and the broad breasted white turkey (BBWT).

Oxide	Bird		
	EBD		BBWT
	Line	Concentration	
Na2O (Sodium oxide)	Ka	0 mg/kg	0.196 Wt %
MgO (Magnesium oxide)	Ka	0.292 Wt %	0.123 Wt %
Al2O3 (Aluminium oxide)	Ka	3.821 Wt %	2.980 Wt %
SiO2 (silicon dioxide)	Ka	35.503 Wt %	32.296 Wt %
P2O5 (Phosphorus Pentoxide)	Ka	5.059 Wt %	3.176 Wt %
SO3 (Sulfur trioxide)	Ka	30.012 Wt %	33.867 Wt %
ClO2 (Chlorine dioxide)	Ka	5.011 Wt %	6.498 Wt %
K2O (Potassium oxide)	Ka	5.673 Wt %	4.162 Wt %
CaO (Calcium oxide)	Ka	12.921 Wt %	15.088 Wt %
TiO2 (Titanium dioxide)	Ka	0.230 Wt %	0.197 Wt %
Cr2O3 (Chromic oxide)	Ka	122 mg/kg	246 mg/kg
Mn2O3 (Manganese oxide)	Ka	242 mg/kg	360 mg/kg
Fe2O3 (Ferric oxide)	Ka	1.358 Wt %	1.234 Wt %
ZnO (Zinc oxide)	Ka	0.063 Wt %	0.100 Wt %
SrO (Strontium oxide)	Ka	201 mg/kg	212 mg/kg

### Ethics statement

All experimental protocols were approved by the Animal Ethical Committee of the Faculty of Veterinary Medicine, Assiut University, Egypt (Approval number: 06/2024/0175). All procedures were performed and conducted in accordance with the relevant guidelines and regulations^38^. All methods are reported in accordance with the ARRIVE guidelines.

## Results

### Gross anatomy

**Fig. 1:**
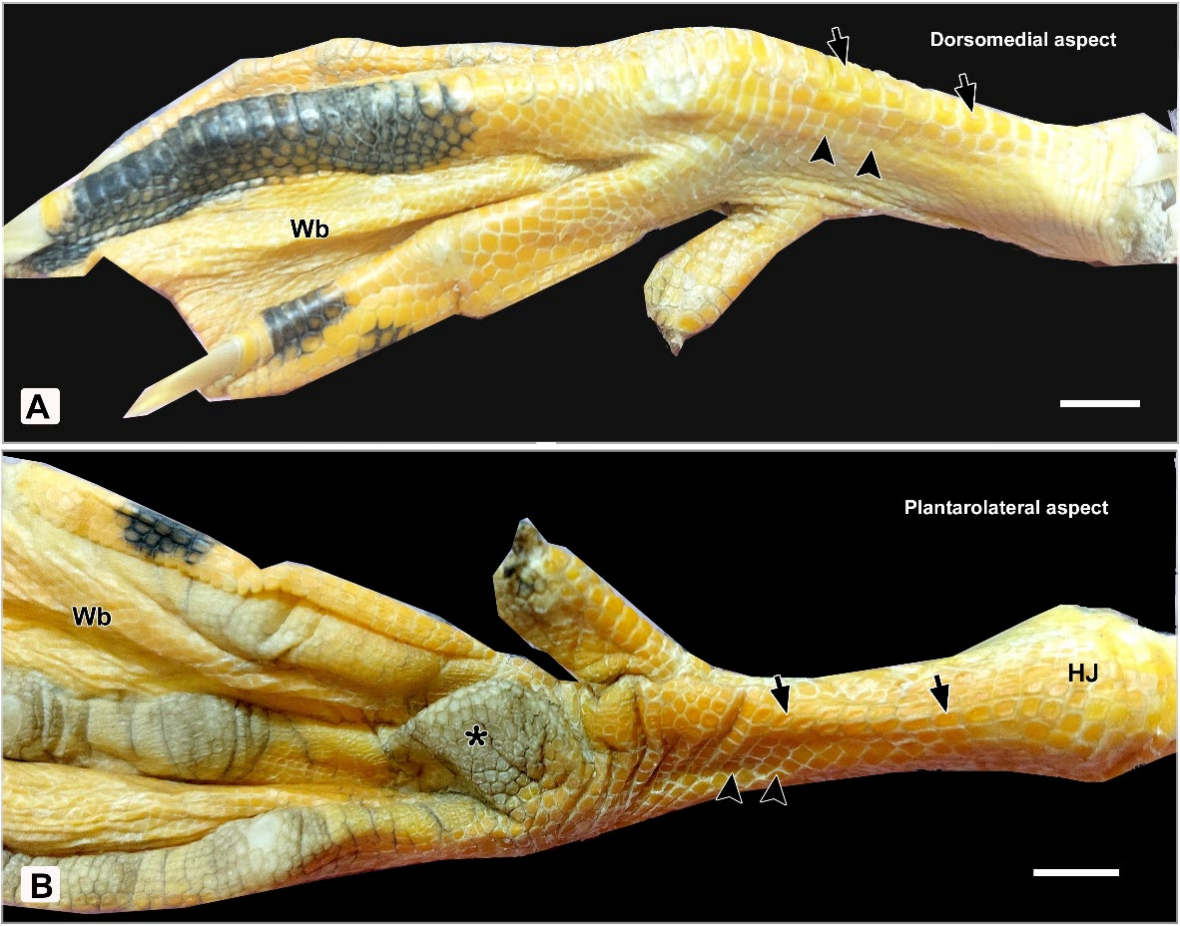
Photographs of the right leg of Egyptian Balady Duck (EBD) showing: (**A**) Dorsomedial, and (**B**) Plantarolateral skin aspects of the shank. Note scute scales (short arrows), scutella scales (arrowheads), hock joint or ankle (HJ), foot or metatarsal pad (asterisk), interdigital web (Wb). Scale bar 1mm.

**Fig. 2:**
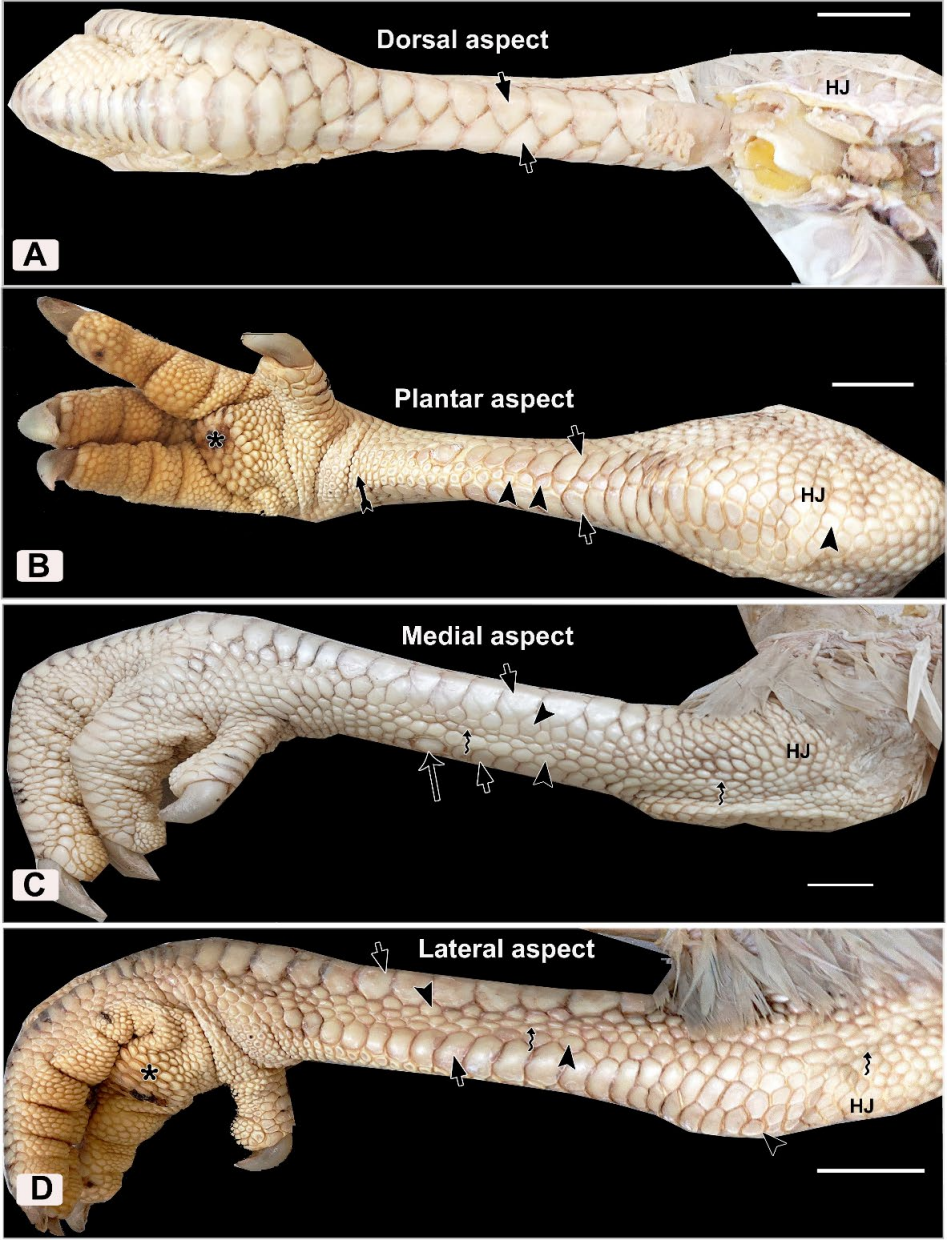
Photographs of the right leg of White Broad Breasted Turkey (WBBT) showing: (**A**) Dorsal, (**B**) Plantar, (**C**) Medial, and (**D**) Lateral skin aspects of the shank. Note scute scales (short arrows), scutella scales (arrowheads), reticulate scales (barbed arrow), interstitial scales (twisted arrows), spur (arrow), hock joint or ankle (HJ), metatarsal pad (asterisks).

The featherless shank skin of the studied birds presented color variations. It was typically yellow to black in Egyptian Balady Duck (EBD) (*Anas boschas domesticus*) (Fig. 1) and creamy-white in Broad Breasted White Turkey (BBWT) (*Meleagris gallopavo*) (Fig. 2). The shank skin (tarsometatarsus) was covered with scales. These scales varied in their types, shapes, sizes, and distributions.

#### Egyptian Balady Duck (EBD)

The scales were distinguished into two types around the shank of EBD: scute and scutella. The scute scales were large, quadrilateral, and rectangular-shaped. They were aligned in 2–3 parallel rows on the dorsal and plantar aspects of the tarsometatarsus, respectively. However, the scutella scales were small, ranging in shape from hexagonal to round or square and located on the medial and lateral aspects. All scales around the shank of EBD were non-overlapped and separated by sulci. The sulci were lighter and paler in color than the scales (Fig. 1A, B).

#### Broad Breasted White Turkey (BBWT)

The scales were categorized into 4 types in WBBT: scute, scutella, reticula, and cancella (interstitial). The scute scales were located on the dorsal and plantar aspects of the shank (Fig. 2A, B). These scales were large, symmetrical hexagonal-shaped, overlapped in a manner resembling the fish scales, and arranged in 2 interlocking rows on the dorsal aspect of the shank (Fig. 2A). However, on the plantar aspect, the scute scales were relatively small, asymmetrical rectangular-shaped, and arranged in 2 parallel rows. The proximal two-thirds of the plantar aspect of the shank revealed a row of round-shaped scutella scales between the scute scales. This row of scales increased in number distally to 2–3 rows and exhibited button-shaped with a central depression (Fig. 2B). The scutella scales were also aligned on the medial and lateral aspects of the tarsometatarsus, close to the scute type. They were variable in size and hexagonal to oval in shape. The reticulate scales were small nodular-shaped, and distinguished on the distal part of the plantar aspect (Fig. 2B). The cancella (interstitial) scales were small, oval to elongate in shape, and arranged in 2 parallel rows among the scutella scales on the lateral and medial surfaces of the tarsometatarsus. These scales were different in sizes and shapes on the entire lateral and medial aspects of the hock joint (Fig. 2C, D).

All scales were non-overlapped and separated by shallow sulci except those on the dorsal aspect of the shank (Fig. 2). The color of sulci was identical to the scales. Overall, the texture of the scaly shank skin of BBWT was harder than that of EBD (Figs. 1, 2).

### Scanning *electron* microscopy

#### Egyptian Balady Duck (EBD)

**Fig. 3:**
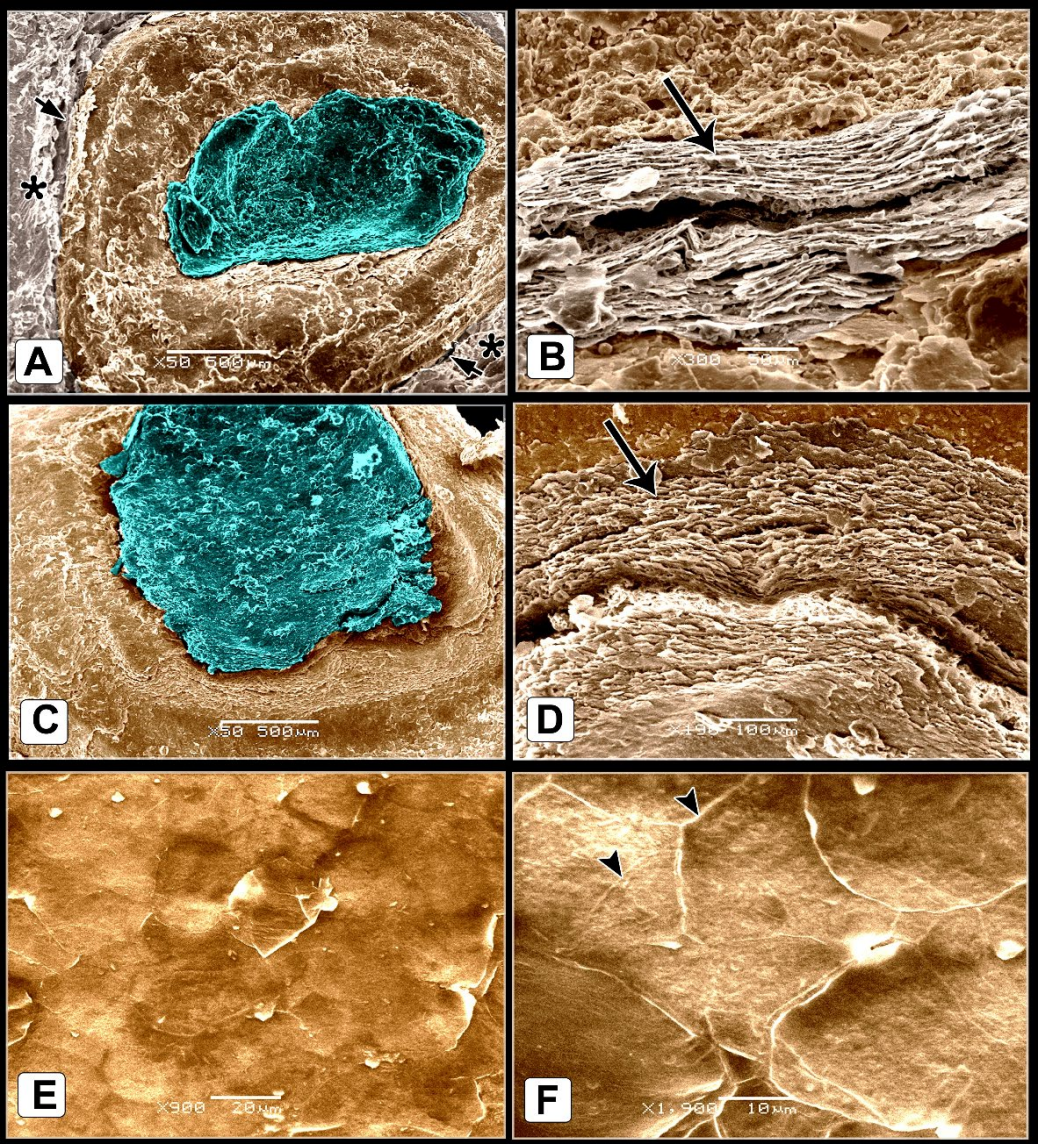
Colored SEM of the dorsal aspect of the shank of EBD showing: (**A**, **C**) Ovoid to quadrilateral-shaped scute scales were characterized by a central depression, the depression covered by an elevated concentric keratinized layer (turquoise color), sulci (short arrows) between the scales revealed an elevated plate-like highly keratinized skin (asterisks). (**B**, **D**) The scales were highly keratinized specifically at the interior areas of the central depression which revealed numerous delicate lamellae of keratin (arrow). (**E**, **F**) The shed (descaled) skin. Note irregular surface due to keratinocytes defining polygonal texture (arrowheads).

**Fig. 4:**
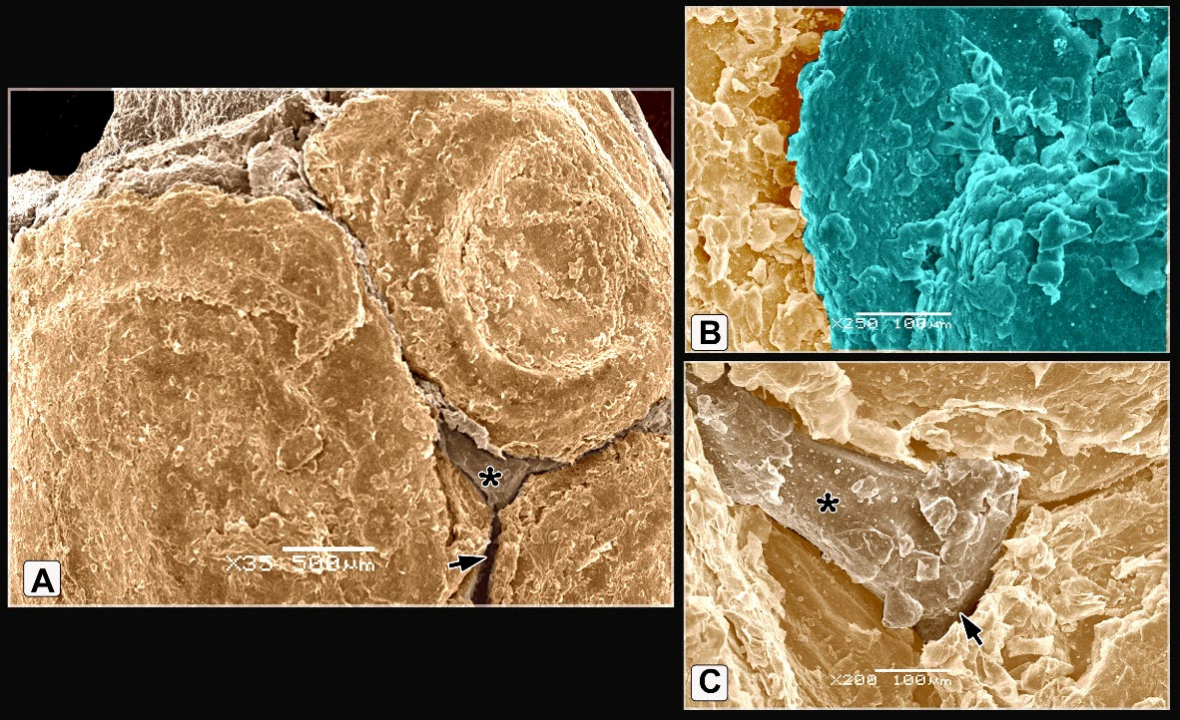
Colored SEM of the plantar aspect of the shank of EBD showing: (A-C) Variable sizes of button-like scute scales, characterized by a central shallow depression and well-defined high thick margins, the depression covered by somewhat an elevated concentric keratinized layer (turquoise color), sulci (short arrows) between the scales revealed a low rod-like highly keratinized skin (asterisk).

The scute scales on the dorsal aspect of the shank were ovoid to quadrilateral-shaped, characterized by a central depression. This depression was covered by an elevated concentric keratinized layer (Fig. 3A, C) and had numerous delicate lamellae of keratin on its interior areas (Fig. 3B, D). The sulci between the scales presented an elevated plate-like, highly keratinized skin (Fig. 3A, C). SEM of the descaled skin revealed an irregular surface due to keratinocytes defining a polygonal texture (Fig. 3E, F). The scute scales on the plantar aspect were button-shaped and variable in size. These scales were distinguished by a shallow central depression and well-defined highly thick margins (Fig. 4A). The depression was covered by a somewhat elevated concentric keratinized layer (Fig. 4B). The sulci revealed a low rod-like keratinized skin (Fig. 4A, C).

**Fig. 5:**
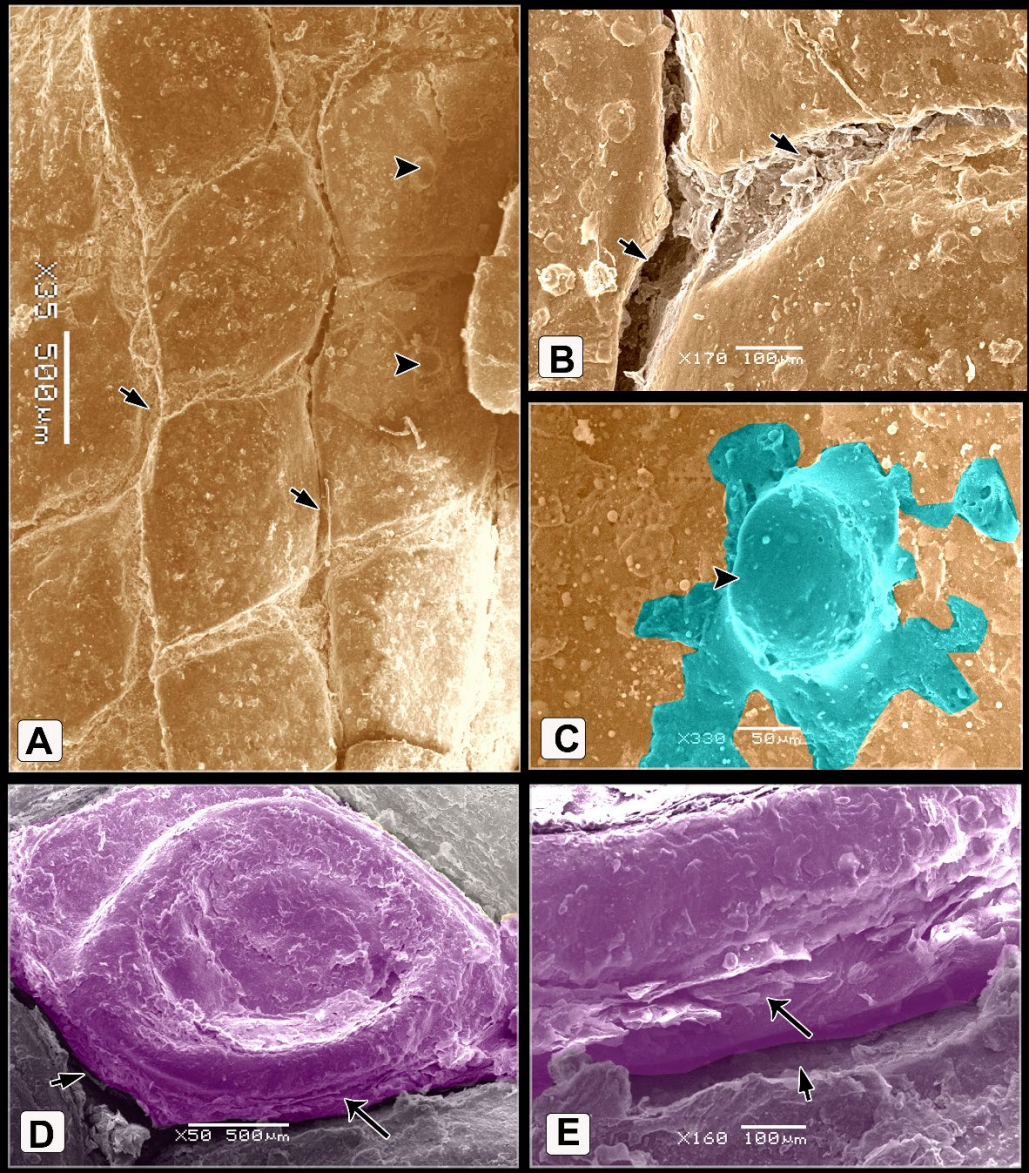
Colored SEM (**A**-**C**) of the medial and (**D**, **E**) of the lateral aspects of the shank of EBD showing: (**A**-**C**) Flat squamous-shaped scutella scales were symmetrically sizes, aligned as bricks-like, sulci (short arrows) between the scales, dome shaped scale sensilla with several procesess (turquoise color; arrowhead). (**D**, **E**) Button-like scutella scales (purple color) with divided keratinized edges (arrows), sulci (short arrow).

**Fig. 6:**
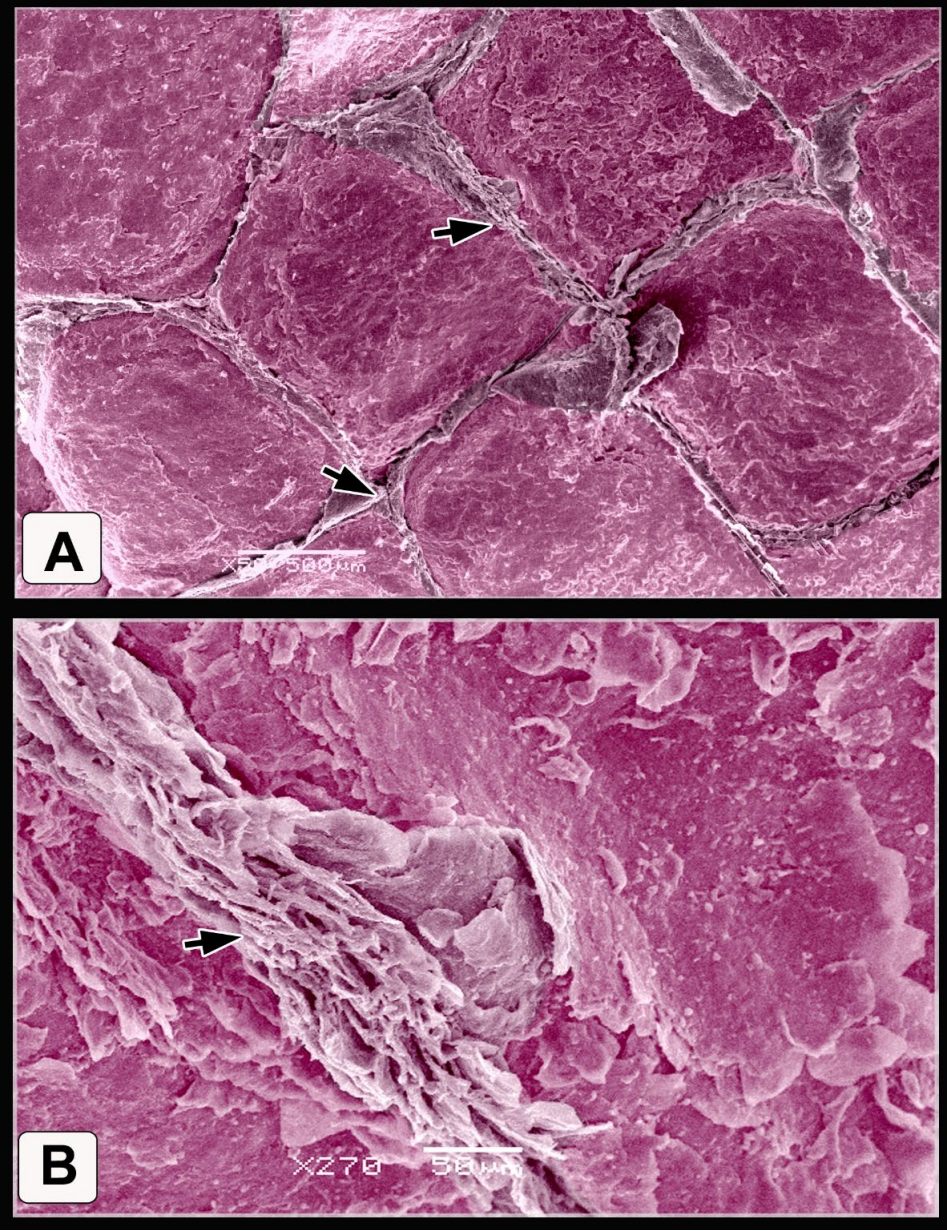
Colored SEM the scaly skin of the ankle (hock joint) of EBD showing: (**A**, **B**) Flat square-shaped scutella scales with prominent lamellae (flakes) of keratin (short arrows) within the sulci between scales.

The scutella scales on the medial aspect were symmetrical flat square-shaped and aligned as bricks-like (Fig. 5A, B). Some scales had dome-shaped scale sensilla with several processes (Fig. 5C). Meanwhile, on the lateral aspect, the button-shaped scutella scales displayed split keratinized edges (Fig. 5D, E). The scales on the ankle (hock joint) resembled those on the medial aspect but with prominent lamellae (flakes) of keratin on the sulci. These lamellae were protruded higher than the surface of the scales (Fig. 6A, B).

#### Broad Breasted White Turkey (BBWT)

**Fig. 7:**
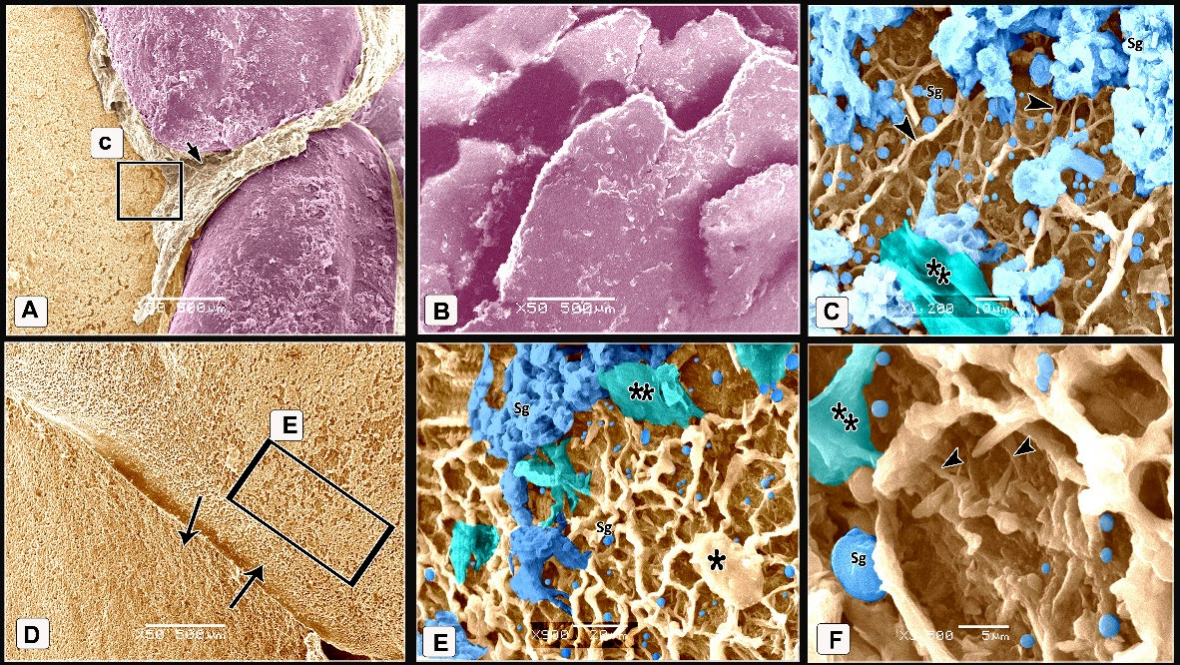
Colored SEM of the dorsal aspect of the shank of WBBT showing: (**A**) Scute scales were demarcated by highly keratinized bands (short arrow). (**B**) The keratin of the scute scales appeared as several irregular layers. (**C**) High magnification of the descaled skin at the area opposite to the sulci (square on image A). Note thin to thick projections (cytoplasmic processes) of the Langerhans cells (arrowheads) were covered with excessive secretory granules (blue color; Sg), keratinocytes (turquoise color; double asterisks). (**D**) Two overlapped plates of the descaled skin (arrows). (**E**, **F**) High magnification of the descaled skin at the site of the overlapping (rectangle on image D). Note Langerhans cell body (asterisk) with abundant long projections (cytoplasmic processes) attached to neighboring keratinocytes (turquoise color; double asterisks), secondary short thin extensions (arrowheads), secretory granules (blue color; Sg).

**Fig. 8:**
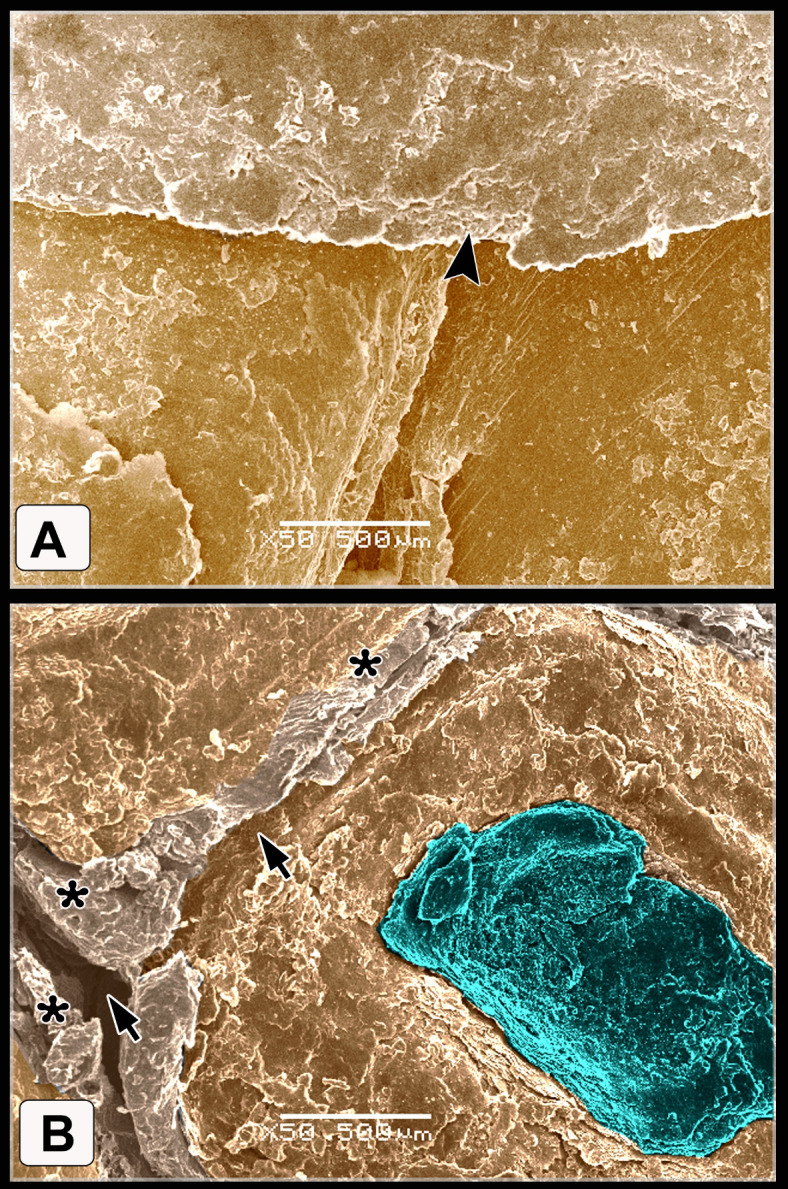
Colored SEM of the plantar aspect of the shank of WBBT showing: (**A**) Flat scute scale with serrated or irregular edges (arrowhead). (**B**) Scutella scale in between the scute scales characterized by a central shallow depression, the depression covered by somewhat an elevated concentric keratinized layer (turquoise color), sulci (short arrows) between the scales revealed a double high rod-like highly keratinized skin (asterisks).

The scales on the dorsal aspect of the shank were demarcated by highly keratinized bands (Fig. 7A). The keratin of the scute scales appeared as irregular layers (Fig. 7B). High magnification of the descaled skin opposite the sulci showed thin to thick projections (cytoplasmic processes) of Langerhans cells (Fig. 7C). These cells were more condensed near the overlapped area of the scales (Fig. 7D-F). Langerhans cells had a large oval-shaped cell body and abundant long projections (cytoplasmic processes). The projections were attached to the neighboring keratinocytes (Fig. 7E), Moreover, these projections were crossed one another resembled a dense network. Each projection displayed secondary short thin extensions (Fig. 7F). Excessive rounded secretory granules coated the Langerhans cells. These granules were aggregated in abundant masses as shown in Figs. 7C, E, and F. The scute scales on the plantar aspect of the shank were flat with serrated or irregular edges (Fig. 8A). The scutella scales between the scute scales displayed a central shallow depression resembled that observed in EBD. The sulci had double high rod-like keratinized skin (Fig. 8B).

**Fig. 9:**
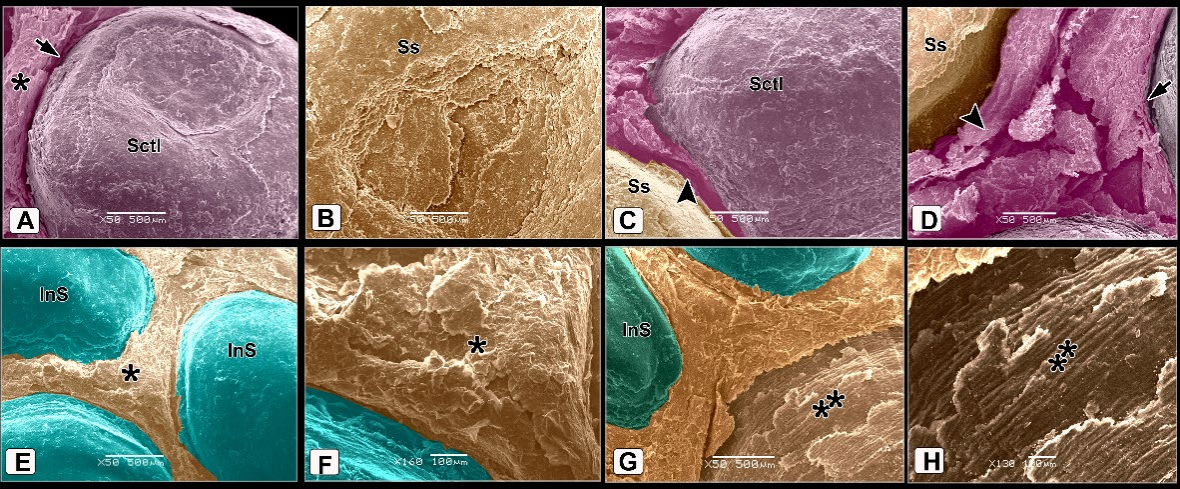
Colored SEM (**A**-**D**) of the medial and (**E**-**H**) of the lateral aspects of the shank of WBBT showing: (**A**-**D**) Scutella scale (Sctl) around the spur had an eccentric shallow depression, sulci (short arrow) with rod of keratin (asterisk), spur scale (Ss) was characterized by concentric flakes of keratin, separated from the surrounding scales by highly keratinized groove (arrowhead). (**E**, **H**) Oval-shaped cancella (interstitial) scales (InS), elevated keratinized skin (asterisk), planned layer of keratin (double asterisks).

**Fig. 10:**
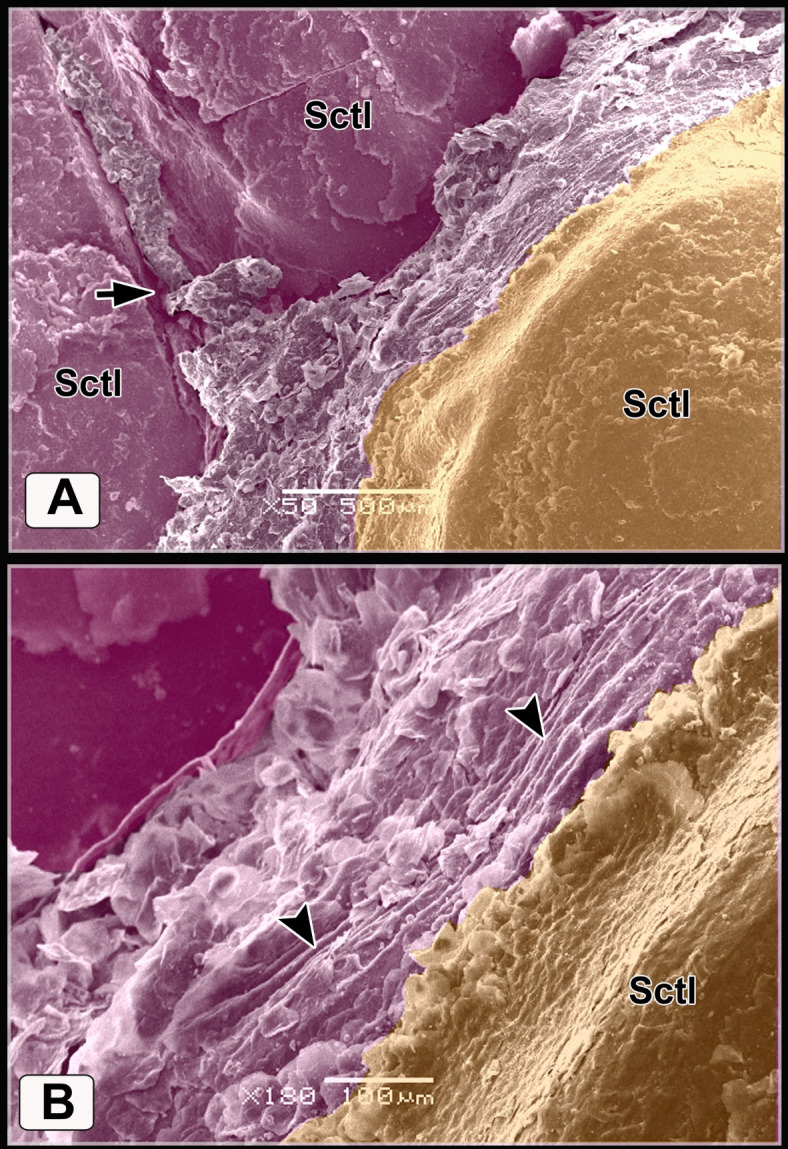
Colored SEM of the scaly skin of the ankle (hock joint) of WBBT showing: (**A**, **B**) Overlapped scutella scales (Sctl), highly keratinized sulcus (short arrow), prominent lamellae (flakes) of keratin on the edges of the scales (arrowheads).

The scutella scales around the spur on the medial aspect had an eccentric shallow depression (Fig. 9A). The spur scale displayed concentric flakes of keratin and separated from the surrounding scales by a highly keratinized groove (Fig. 9B-D). The oval-shaped interstitial scales were embedded in the keratinized skin. A planned layer of keratin appeared near those scales (Fig. 9E-H). Some scutella scales on the ankle had prominent lamellae (flakes) of keratin on their edges with highly keratinized sulci (Fig. 10A, B).

### Light microscopy

**Fig. 11:**
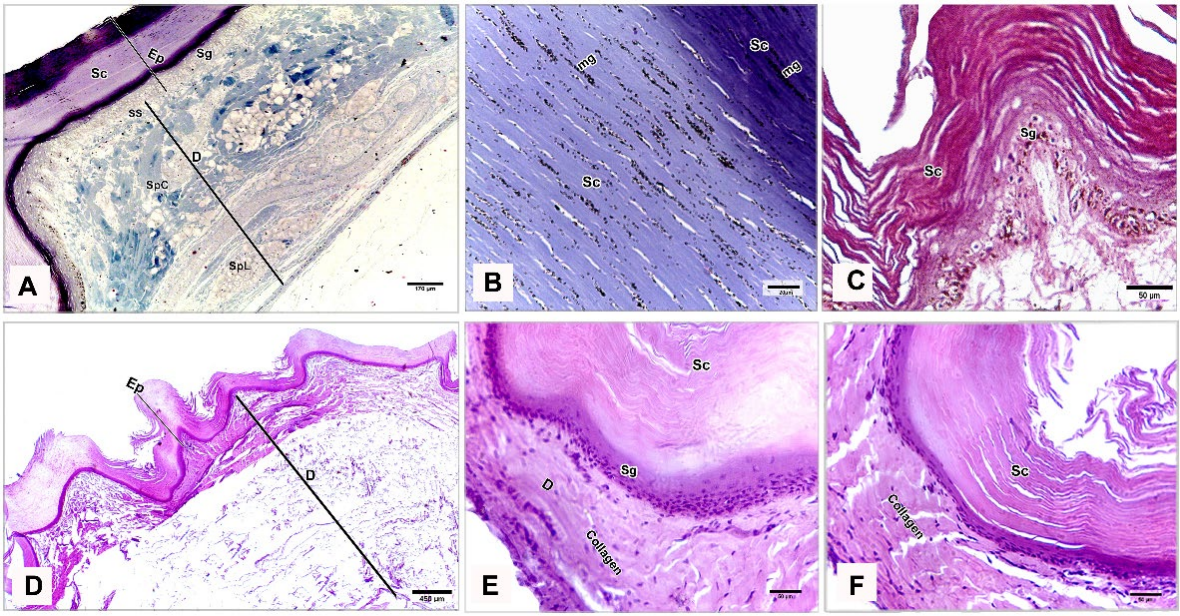
Semithin sections (**A**, **B**) stained by toluidine blue, paraffin section (**C**) stained by H&E of the dorsal skin aspect and paraffin sections (**D**-**F**) of the plantar skin aspect of the shank of EBD showing: Epidermis (Ep) was represented by stratum corneum (Sc), and stratum germinativum (Sg), melanin granules (mg), dermis (**D**) was composed of stratum superficiale (SS), stratum compactum (SpC) and stratum laxum (SpL) of stratum profundum, collagen fibers (collagen) parallel to the epidermis and occasionally perpendicular fibers.

The shank skin consisted of an outer, nonvascular, epithelial layer (epidermis) and an inner, deeper connective tissue layer, the corium (dermis). The epidermis was relatively thinner than the dermis, represented by two main cell layers: a non-living cellular (stratum corneum) and a living cellular (stratum germinativum). The stratum germinativum was made up of three layers from inside to outside: the stratum basale (basal layer), the stratum spinosum (stratum intermedium), and the outermost stratum transitivum (transitional layer). The dermis was composed of two principal layers: stratum superficiale (superficial layer) and stratum profundum (deep layer) which represented the stratum compactum and the stratum laxum (Fig. 11A).

#### Egyptian Balady Duck (EBD)

**Fig. 12:**
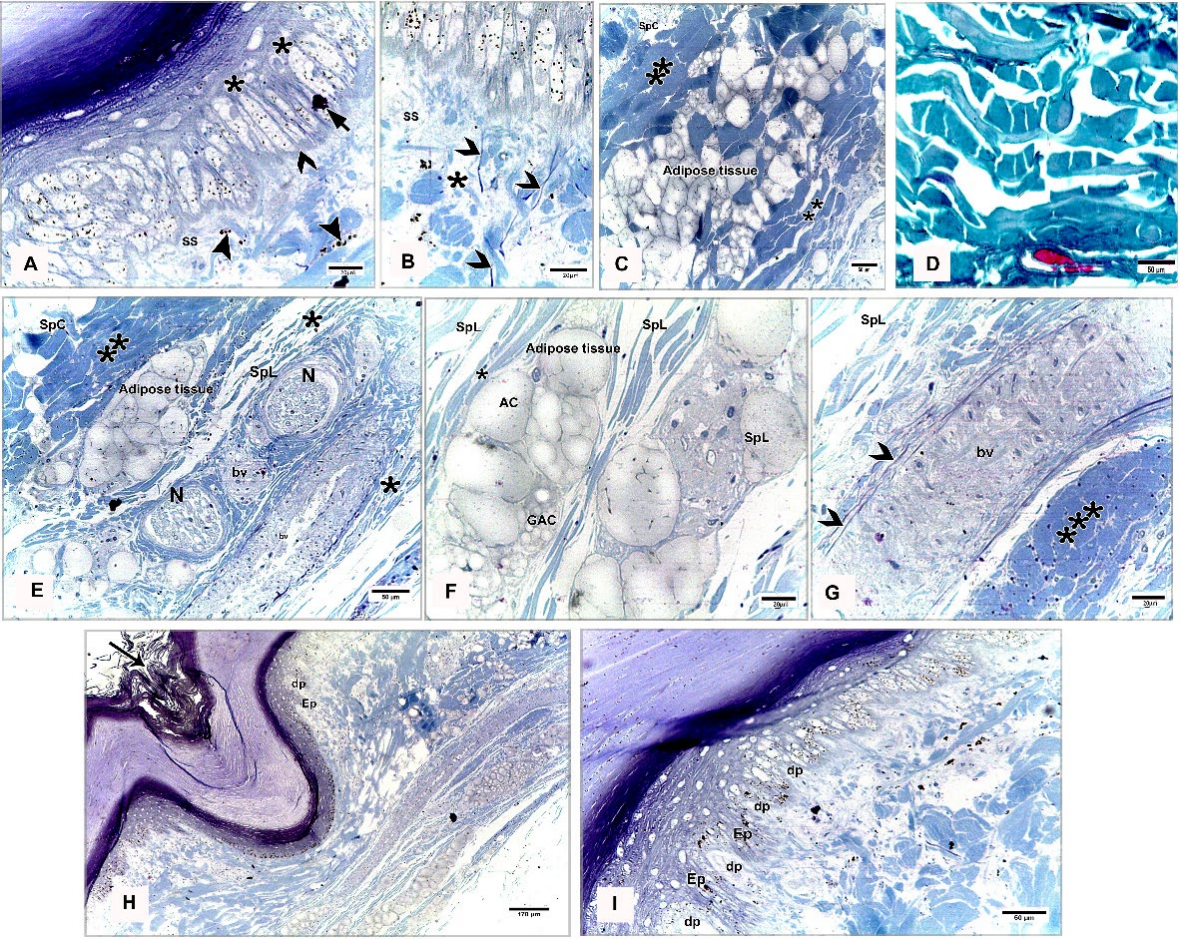
Semithin sections (A-C&E-I) stained by toluidine blue of the dorsal skin aspect and paraffin section (D) stained by Masson’s trichrome of the plantar skin aspect of the shank of EBD showing: (**A**) Columnar basal cells (asterisks) undergo hypertrophy, with pale vacuolated cytoplasm, contained melanin granules rested on the basement membrane (forked arrowhead), melanocytes (short arrow) interspersed between the basal cells, melanin pigment (arrowheads) within the stratum superficiale (SS). (**B**) Numerous telocytes (forked arrowheads) within the irregular collagen fibers (asterisk) at stratum superficiale (SS). (**C**) Adipose tissue within the dense regular collagen fibers (double asterisks) at the stratum compactum (SpC). (**D**) Bundles of the collagen fibers stained by Masson’s trichrome. (**E**–**G**) Dense regular collagen fibers (double asterisks) and adipose tissue at the stratum compactum (SpC), loose regular collagen fibers (asterisks) at the stratum laxum (SpL) contained nerve endings (N), large blood vessels (bv), excessive adipose tissue, formed of variable sizes of adipocytes (AC) with growing cells (GAC), abundant telocytes (forked arrowheads) close to blood vessels, dense collagen fibers (triple asterisks). (**H**, **I**) Near the sulcus region (arrow) between the scales, epidermal pegs (Epg) interdigitated with the dermal connective tissue papillae (dp).

**Fig. 13:**
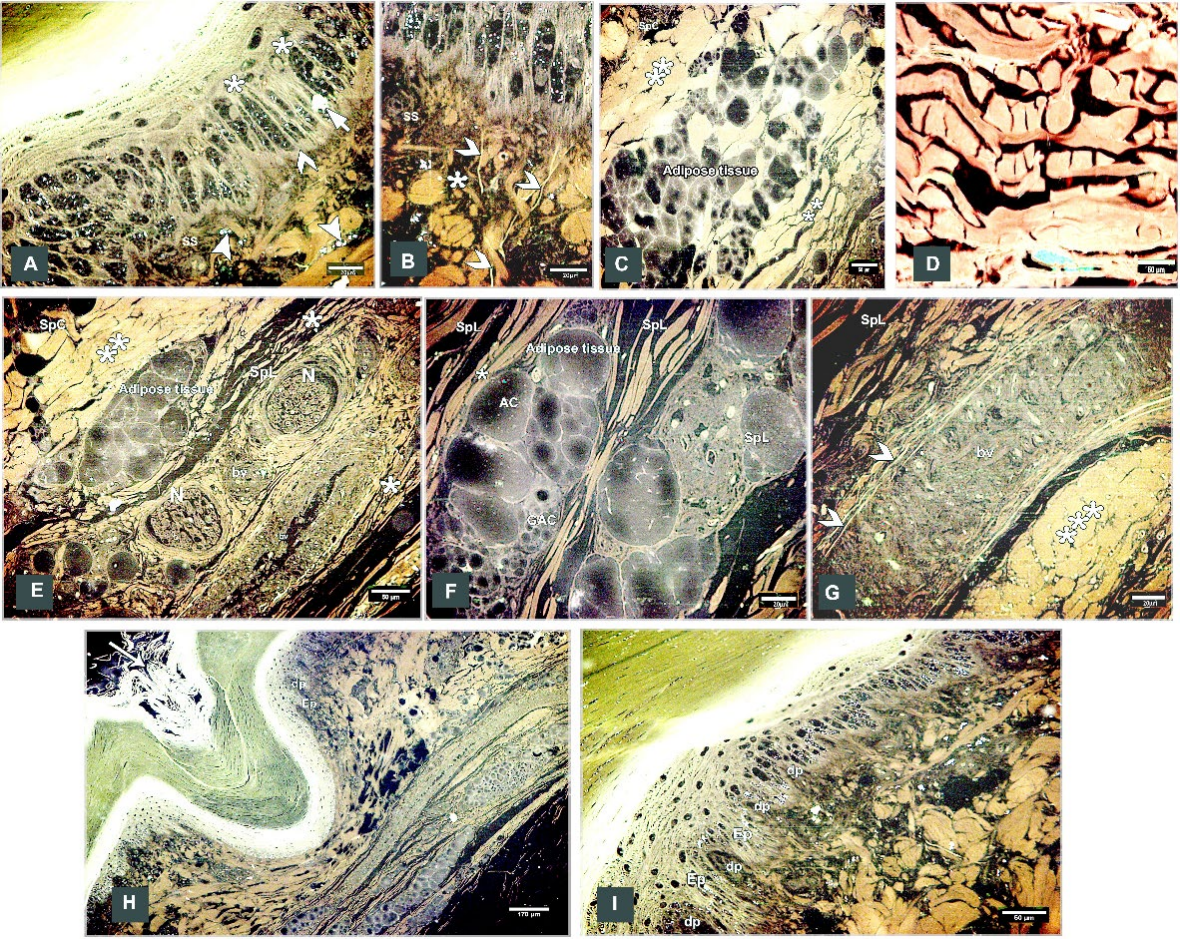
Negative image of the Fig. 12 to clarify the semithin sections (A-C&E-I) stained by toluidine blue of the dorsal skin aspect and paraffin section (D) stained by Masson’s trichrome of the plantar skin aspect of the shank of EBD. (**A**) Columnar basal cells (asterisks) undergo hypertrophy, with pale vacuolated cytoplasm, contained melanin granules rested on the basement membrane (forked arrowhead), melanocytes (short arrow) interspersed between the basal cells, melanin pigment (arrowheads) within the stratum superficiale (SS). (**B**) Numerous telocytes (forked arrowheads) within the irregular collagen fibers (asterisk) at stratum superficiale (SS). (**C**) Adipose tissue within the dense regular collagen fibers (double asterisks) at the stratum compactum (SpC). (**D**) Bundles of the collagen fibers stained by Masson’s trichrome. (**E**–**G**) Dense regular collagen fibers (double asterisks) and adipose tissue at the stratum compactum (SpC), loose regular collagen fibers (asterisks) at the stratum laxum (SpL) contained nerve endings (N), large blood vessels (bv), excessive adipose tissue, formed of variable sizes of adipocytes (AC) with growing cells (GAC), abundant telocytes (forked arrowheads) closed to blood vessels, dense collagen fibers (triple asterisks). (**H**, **I**) Near the sulcus region (arrow) between the scales, epidermal pegs (Epg) interdigitated with the dermal connective tissue papillae (dp).

At the dorsal and plantar skin, the stratum corneum was a thick compact layer. It consisted of several strata of superficial flattened keratinized cells without stainable nuclei, occupied by excessive melanin granules (Fig. 11B). The keratin layer at or near sulci was corrugated (Fig. 11C-F). The cells of the stratum germinativum were basophilic stratified squamous type with centrally located large oval nuclei except those of the transitional layer, which were acidophilic (Fig. 11E). The basal cell layer of the stratum germinativum was the deepest epidermal layer, filled with melanin granules (Fig. 11C). Some basal columnar cells with pale vacuolated cytoplasm resting on the basement membrane at the epidermal hinge (sulci). These cells contained excessive melanin granules and undergoing hypertrophic, which formed a hypertrophic stratum. The melanocytes were interspersed between the basal cells (Figs. 12A & 13A).

The stratum superficiale of the dermis was a thin layer, formed of loose irregular collagen fibers with small blood vessels, fibroblasts, telocytes, and melanin pigments (Figs. 12A, B & 13A, B). The stratum profundum compactum was characterized by thick dense regular collagen fibers with large blood vessels, numerous fibroblasts, and adipose tissue (Figs. 12C, D & 13C, D). The collagen bundles were aligned parallel to the epidermis. Perpendicular fibers were observed especially at the sulcus area (Fig. 11F). The stratum profundum laxum consisted of loosely arranged connective tissue with delicate regular and irregular collagen fibers, large blood vessels, and excessive adipose tissue which formed of variable-sized adipocytes and growing fat cells (Figs. 12E, F & 13E, F). Abundant telocytes and dense collagen fibers were detected close to the blood vessels (Figs. 12G & 13G). The epidermal pegs interdigitated with the dermal connective tissue papillae were observed near the sulcus area (Figs. 12H, I & 13H, I).

**Fig. 14:**
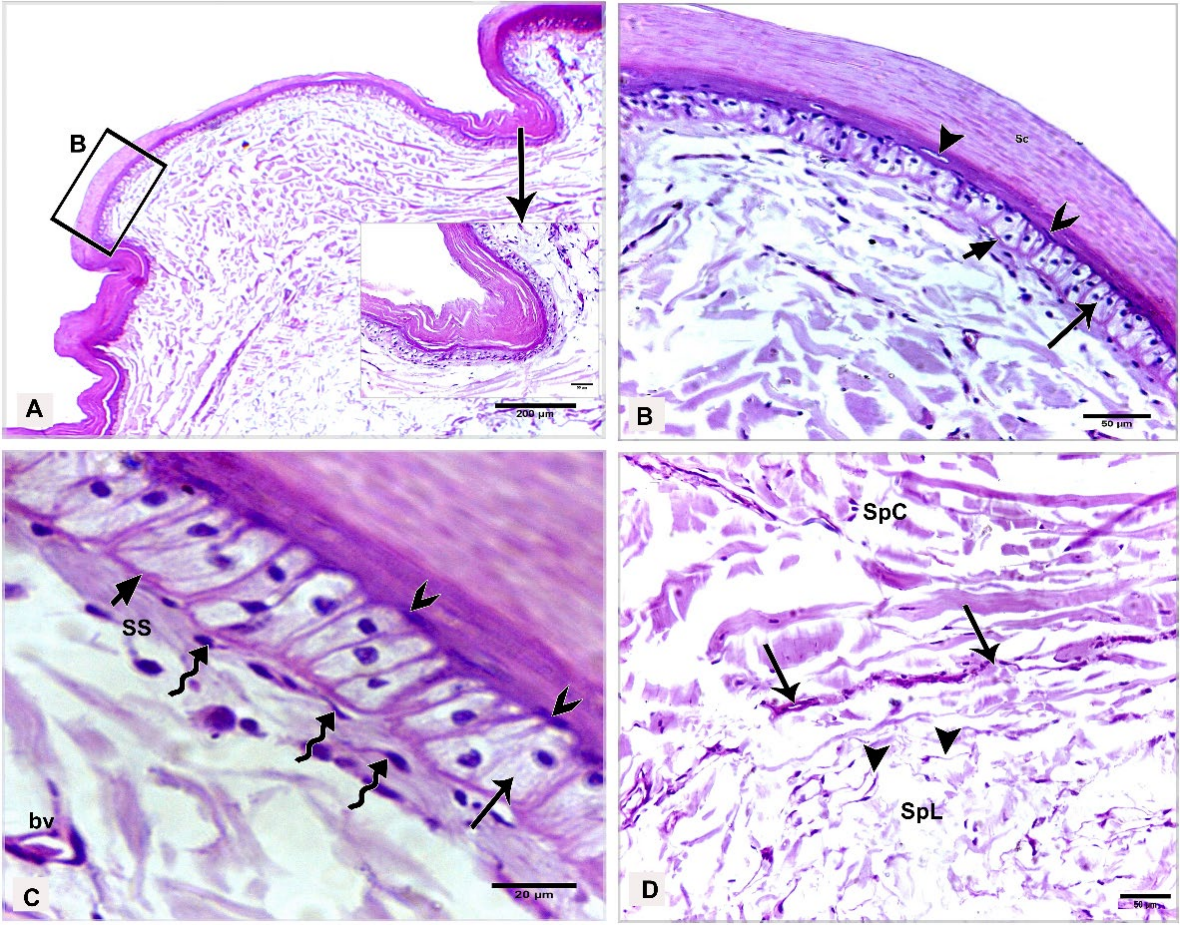
Paraffin sections (**A**-**D**) stained by H&E of the medial skin aspect of the shank of EBD showing: (**A**-**C**) Stratum corneum (Sc), a wavy keratin layer at the hinge epidermis (sulci) as shown in image A), stratum germinativum possessed of a single layer of tall columnar cells (arrow) with rounded centrally located nuclei and vacuolated cytoplasm, laying on the basement membrane (short arrow), the stratum spinosum was represented by a thin layer of flattened basophilic cells (forked arrowheads), stratum transitivum formed of few scattered, flattened vacuolated cells (arrowhead) with degenerating (pyknotic) nuclei, numerous fibroblast cells (twisted arrows) close to the basement membrane within the stratum superficiale (SS) with delicate blood vessels (bv). (**D**) Thin line of blood vessels (arrows) between stratum compactum (SpC) and stratum laxum (SpL), telocytes with long thick and thin telopodes (arrowheads).

**Fig. 15:**
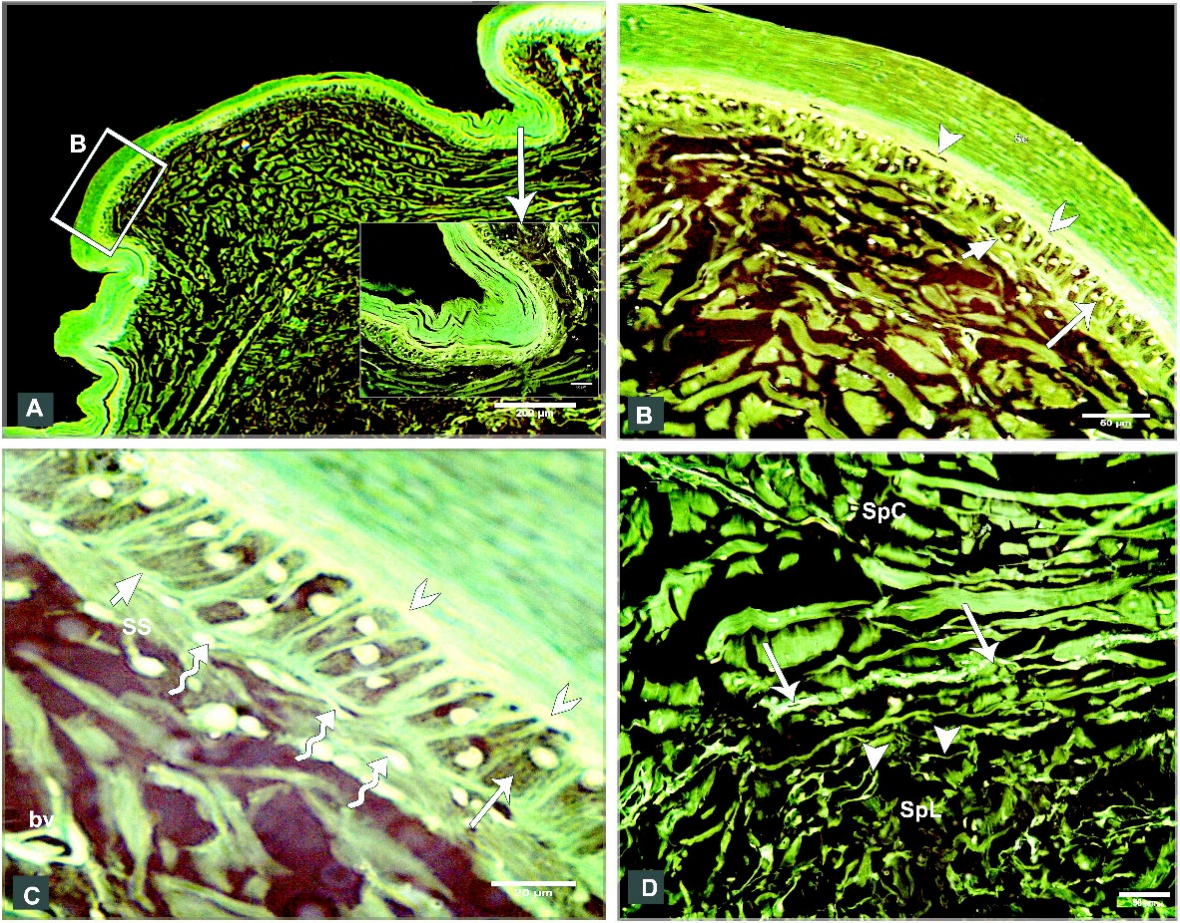
Negative image of the Fig. 14 to clarify the paraffin sections (**A**-**D**) stained by H&E of the medial skin aspect of the shank of EBD. (**A**-**C**) Stratum corneum (Sc), a wavy keratin layer at the hinge epidermis (sulci) as shown in image A), stratum germinativum possessed of a single layer of tall columnar cells (arrow) with rounded centrally located nuclei and vacuolated cytoplasm, laying on the basement membrane (short arrow), the stratum spinosum was represented by a thin layer of flattened basophilic cells (forked arrowheads), stratum transitivum formed of few scattered, flattened vacuolated cells (arrowhead) with degenerating (pyknotic) nuclei, numerous fibroblast cells (twisted arrows) close to the basement membrane within the stratum superficiale (SS) with delicate blood vessels (bv). D) Thin line of blood vessels (arrows) between stratum compactum (SpC) and stratum laxum (SpL), telocytes with long thick and thin telopodes (arrowheads).

At the medial and lateral skin, the stratum corneum was thinner and straighter (Figs. 14A & 15A). The stratum basale of the stratum germinativum exhibited a single layer of tall columnar cells with rounded centrally located nuclei and a vacuolated cytoplasm, laying on the basement membrane. This membrane was distinct, made up collagen fibers and located between the plasma membrane of the basal cells and the dermis (Figs. 14B, C & 15B, C). The stratum spinosum was represented a thin layer of flattened basophilic cells (Figs. 14B, C & 15B, C). The stratum transitivum was composed of a few scattered, flattened vacuolated cells with degenerating (pyknotic) nuclei in which the final processes of keratinization were taken place (Figs. 14B & 15B). The hinge epidermis (sulci) was more deeply stained areas and presented a wavy keratin layer (Figs. 14A & 15A).

The stratum superficiale was similar to that of the dorsal and plantar aspects. As well as it contained numerous fibroblast cells close to the basement membrane and basal cells layer of the epidermis (Figs. 14C & 15C). The stratum profundum compactum was characterized by a regular arrangement of wavy dense collagen fibers parallel to the epidermis. This layer was marked from the stratum profundum laxum by a thin layer of small blood vessels (Figs. 14D & 15D). The stratum laxum showed a low density of irregular collagen fibers and abundant telocytes which were identified near the capillary layer between the dermal deep layers. Telocytes were distinguished by their cell prolongations (thick and thin telopodes) (Figs. 14D & 15D).

#### Broad Breasted White Turkey (BBWT)

**Fig. 16:**
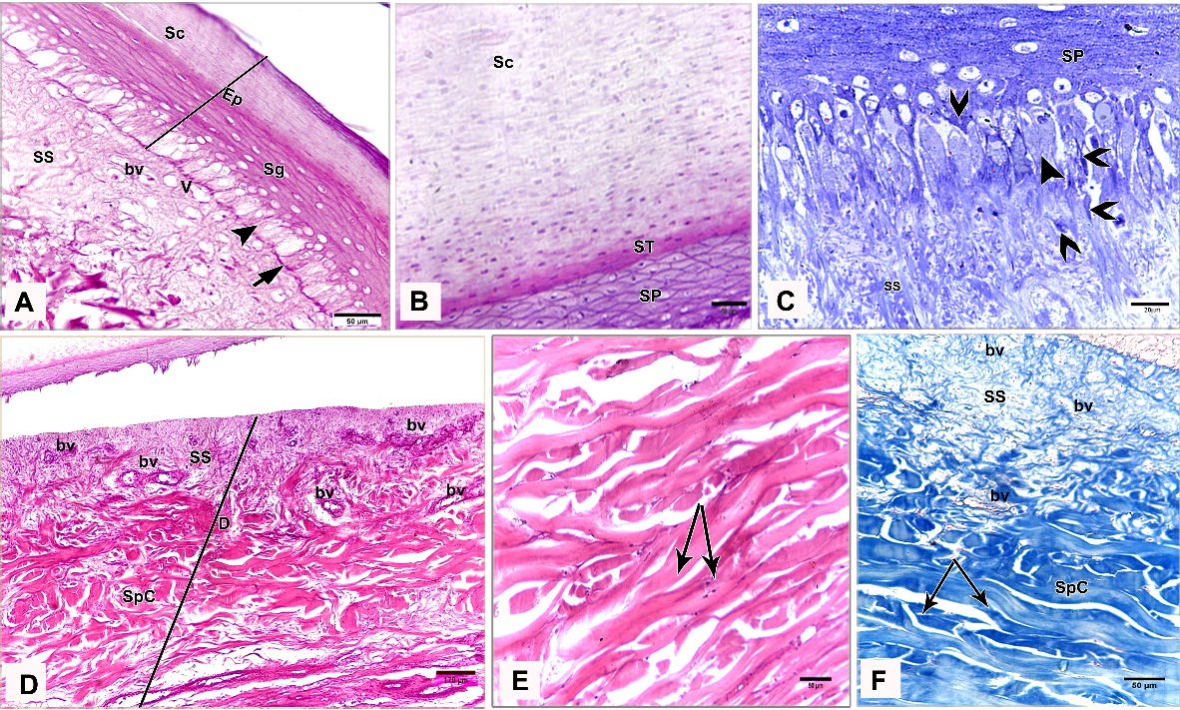
Paraffin sections (**A**, **B**, **D**, **E**) stained by H&E, (**F**) stained by Masson’s trichrome and semithin section (**C**) stained by toluidine blue of the dorsal skin aspect (**A**, **C**, **F**) and of the plantar skin aspect (**B**, **D**, **E**) of the shank of BBWT showing: (**A**, **B**) Epidermis (Ep) was represented by stratum corneum (Sc) consisted of several layers of flattened keratinized cells with distinct stainable elongated nuclei, and stratum germinativum (Sg) was composed of basal columnar cells with intracellular vacuoles (arrowhead) resting on the basement membrane (short arrow), stratum spinosum (SP) was arranged in 6–7 layers of large, polyhedral shaped cells, stratum transitivum (ST) was formed of flattened basophilic cells, connective tissue stratum superficiale (SS) contain blood vessels (bv), different sizes of vacuoles (V) just underneath the basement membrane. (**C**) Stratum spinosum (SP), Langerhans cells (forked arrowheads) were interspersed between the basal cells (arrowhead) and within collagen fibers in stratum superficiale (SS). Note basal cells were columnar type filled with lipid droplets and Langerhans cells had deeply stained cytoplasm and had a cell body and cell processes. (**D**-**F**) Stratum superficiale (SS) of dermis (**D**) formed of crossed-linked loose irregular collagen fibers and abundant blood vessels (bv) that demarcated from stratum compactum (SpC) of stratum profundum which was represented by parallel bundles of collagen fibers (arrows) with blood vessels (bv).

**Fig. 17:**
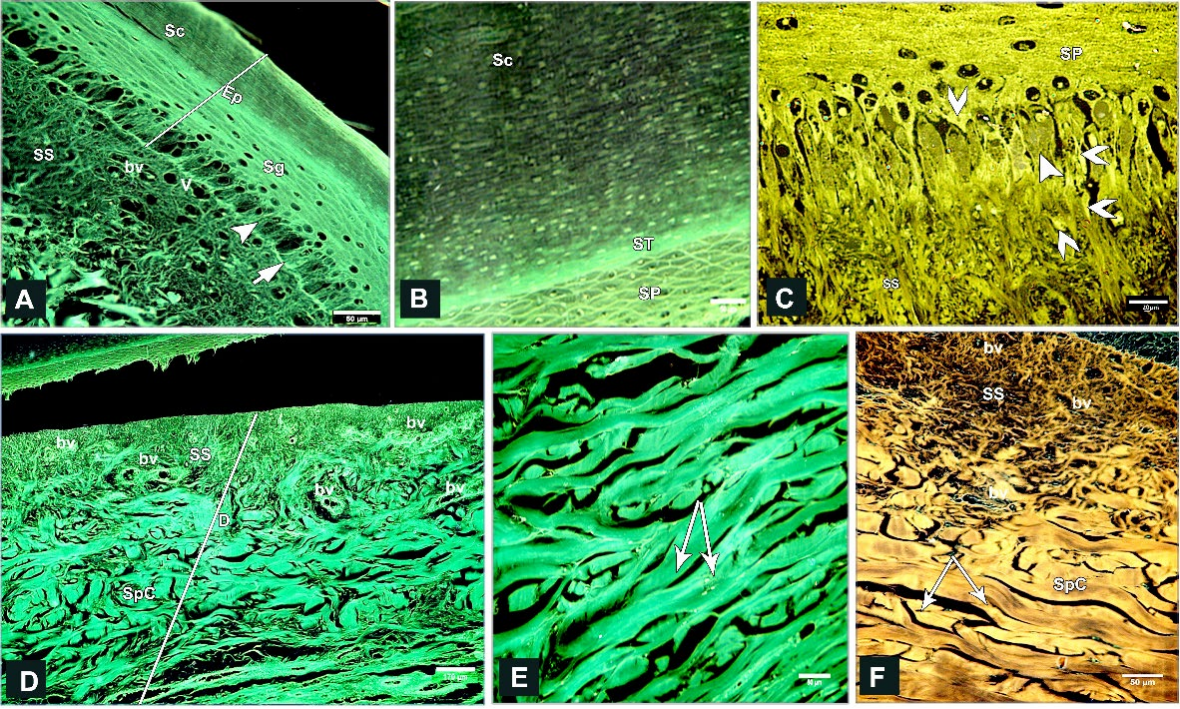
Negative image of the Fig. 14 to the paraffin sections (**A**, **B**, **D**, **E**) stained by H&E, (**F**) stained by Masson’s trichrome and semithin section (**C**) stained by toluidine blue of the dorsal skin aspect (**A**, **C**, **F**) and of the plantar skin aspect (**B**, **D**, **E**) of the shank of BBWT. (**A**, **B**) Epidermis (Ep) was represented by stratum corneum (Sc) consisted of several layers of flattened keratinized cells with distinct stainable elongated nuclei, and stratum germinativum (Sg) was composed of basal columnar cells with intracellular vacuoles (arrowhead) resting on the basement membrane (short arrow), stratum spinosum (SP) was arranged in 6–7 layers of large, polyhedral shaped cells, stratum transitivum (ST) was formed of flattened basophilic cells, connective tissue stratum superficiale (SS) contain blood vessels (bv), different sizes of vacuoles (V) just underneath the basement membrane. (**C**) Stratum spinosum (SP), Langerhans cells (forked arrowheads) were interspersed between the basal cells (arrowhead) and within collagen fibers in stratum superficiale (SS). Note basal cells were columnar type filled with lipid droplets and Langerhans cells had deeply stained cytoplasm and had a cell body and cell processes. (**D**-**F**) Stratum superficiale (SS) of dermis (**D**) formed of crossed-linked loose irregular collagen fibers and abundant blood vessels (bv) that demarcated from stratum compactum (SpC) of stratum profundum which was represented by parallel bundles of collagen fibers (arrows) with blood vessels (bv).

At the dorsal and plantar skin, the epidermal stratum corneum was an extremely thick compact layer and consisted of several layers of flattened keratinized cells with distinct stainable elongated nuclei (Figs. 16A, B & 17A, B). The basal layer of the stratum germinativum consisted of columnar cells with intracellular vacuoles resting on the basement membrane (Figs. 16A & 17A). By semithin sections, these cells were filled with lipid droplets (Figs. 16C & 17C). Langerhans cells were interspersed between the basal layer cells and observed within the collagen fibers of the stratum superficiale as well. Langerhans cells had a cell body with deeply stained cytoplasm and cell processes (Figs. 16C & 17C). The cells of the stratum spinosum were arranged in 6–7 layers of large polyhedral-shaped cells with spherical centrally located nuclei. Furthermore, the cells of the stratum transitivum were flattened basophilic (Figs. 16B & 17B).

In the dermis, the stratum superficiale consisted of dense connective tissue with cross-linked loose irregular collagen fibers, abundant small blood vessels, and vacuoles just underneath the basement membrane. Some irregular collagen fibers were prolonged toward the epidermis. A layer of blood vessels was positioned parallel to the epidermis, marking the boundary between the stratum superficiale and stratum compactum. The blood vessels were numerous at the plantar skin (Figs. 16D & 17D).

**Fig. 18:**
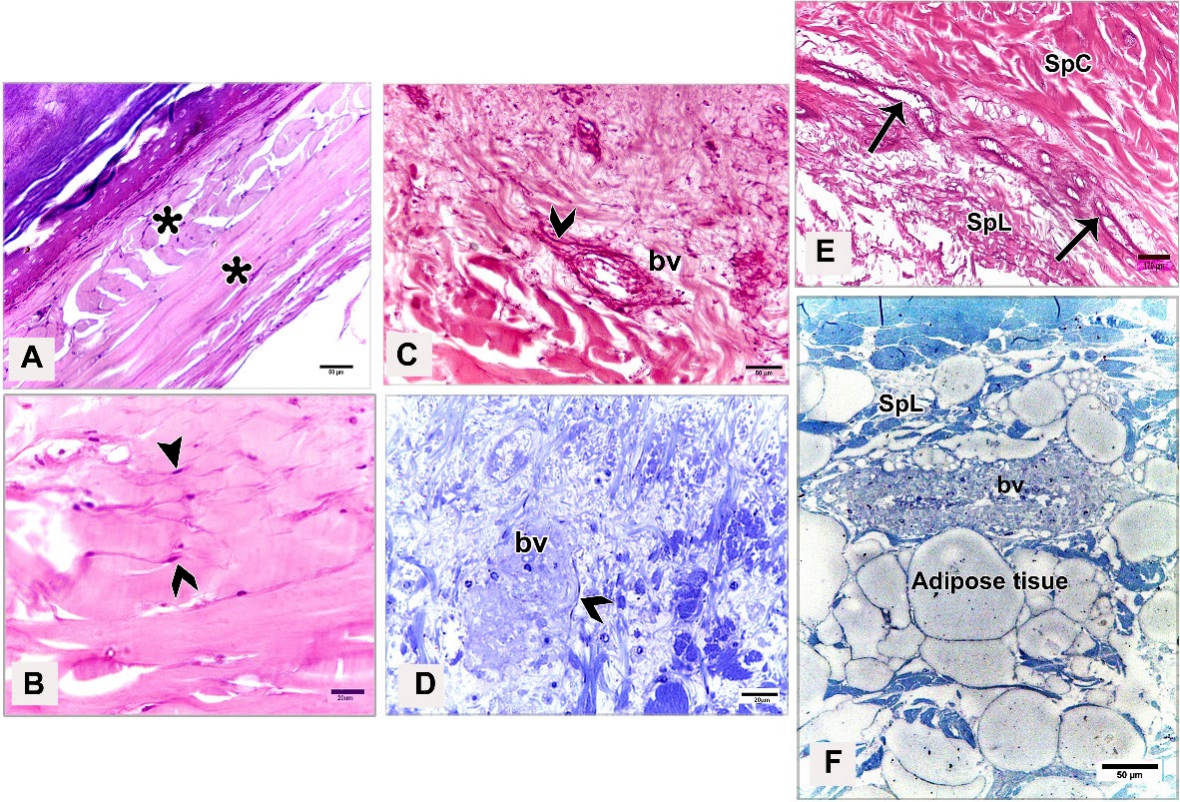
Paraffin sections (**A**, **B**, **C**, **E**) stained by H&E of the plantar skin aspect and semithin sections (**D**, **F**) stained by toluidine blue of the dorsal skin aspect of the shank of BBWT showing: (**A, B**) Variable arrangements of the dense regular thick collagen (asterisks) at the hinge epidermal region, numerous fibroblasts (arrowhead) and telocytes (forked arrowhead). (**C**, **D**) Telocytes (forked arrowheads) closed to blood vessels (bv). (**E**–**F**) Stratum laxum (SpL) of the stratum profundum with thin collagen fibers and adipose tissue and blood vessles (bv), demarcated from the stratum compactum (SpC) by a layer of blood vessels termed deep capillary (arrows).

The stratum profundum compactum was represented by bundles of dense regular collagen fibers, which were thicker and more compact at the dorsal skin (Figs. 16E, F & 17E, F). Numerous fibroblasts and telocytes were recognized within the collagen fibers (Fig. 18A, B). Telocytes were also detected near the blood vessels in both dermal layers (Fig. 18C, D). The stratum profundum laxum was characterized by thin collagen fibers, large blood vessels, and adipose tissue. It was demarcated from the stratum compactum by a layer of blood vessels termed deep capillary (Fig. 18E, F).

**Fig. 19:**
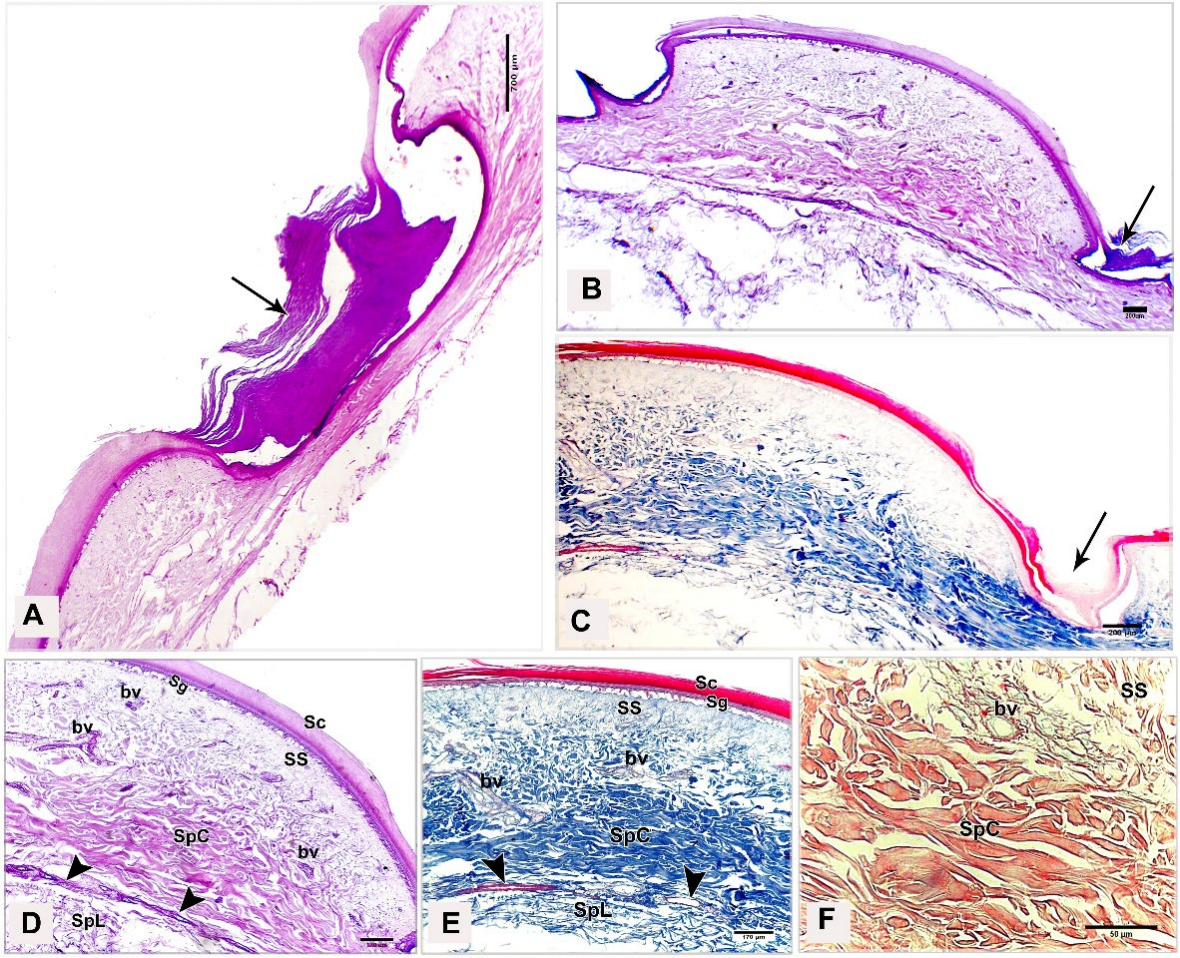
Paraffin sections (**A**, **B**, **D**) stained by H&E, (**F**) stained by Picrosirius red of the medial skin aspect, and (**C**, **E**) stained by Masson’s trichrome of the lateral skin aspect of the shank of BBWT showing: (**A-F**) Sulci regions or epidermal hinge (arrow), stratum corneum (Sc), stratum germinativum (Sg), stratum superficiale (SS), less density of the collagen fibers of the dermis with less blood vessels (bv), and deep capillary row (arrow heads) between stratum compactum (SpC) and stratum laxum (SpL).

**Fig. 20:**
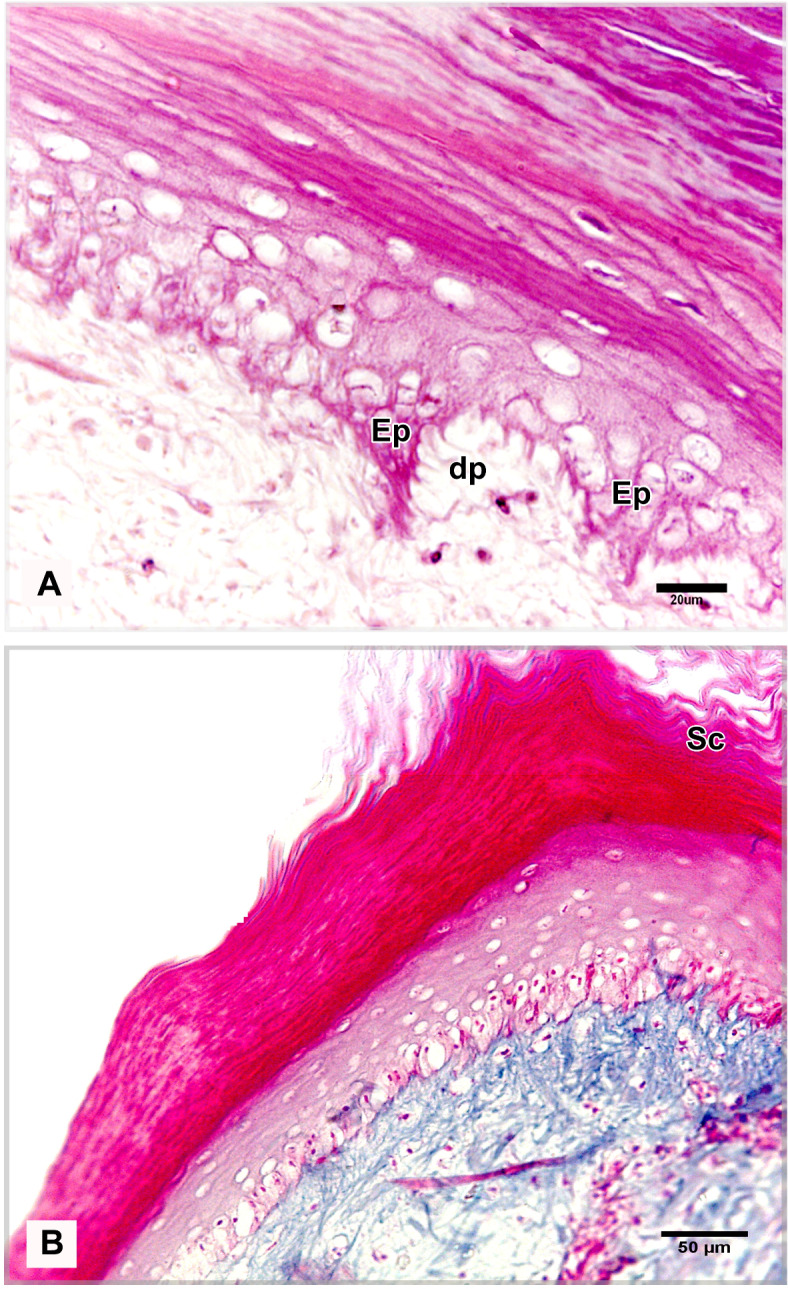
Paraffin sections (**A**) stained by H&E of the medial skin aspect and (B) stained by Masson’s trichrome of the lateral skin aspect of the shank of BBWT showing: A) Few epidermal pegs (Epg) interdigitated with dermal connective tissue papillae (dp) were observed near the epidermal hinge (sulci areas). (**B**) Wavy keratin layer (Sc) was noticed at the epidermal hinge.

At the medial and lateral skin, the histological findings of the epidermal and dermal layers were approximately similar to those of the dorsal and plantar skin but with some variations: a thinner stratum corneum, fewer collagen fibers in the dermis, less vascularized dermis, (Fig. 19A-F) as well as fewer delicate dermal papillae, and a wavy keratin layer near the hinge epidermis (Fig. 20A, B).

### Energy-dispersive X ray Fluorescence (ED-XRF) analysis

The elemental composition of the scaly shank skin of EBD and BBWT was analyzed, along with gross, SEM, and histological investigations.

**Fig. 21:**
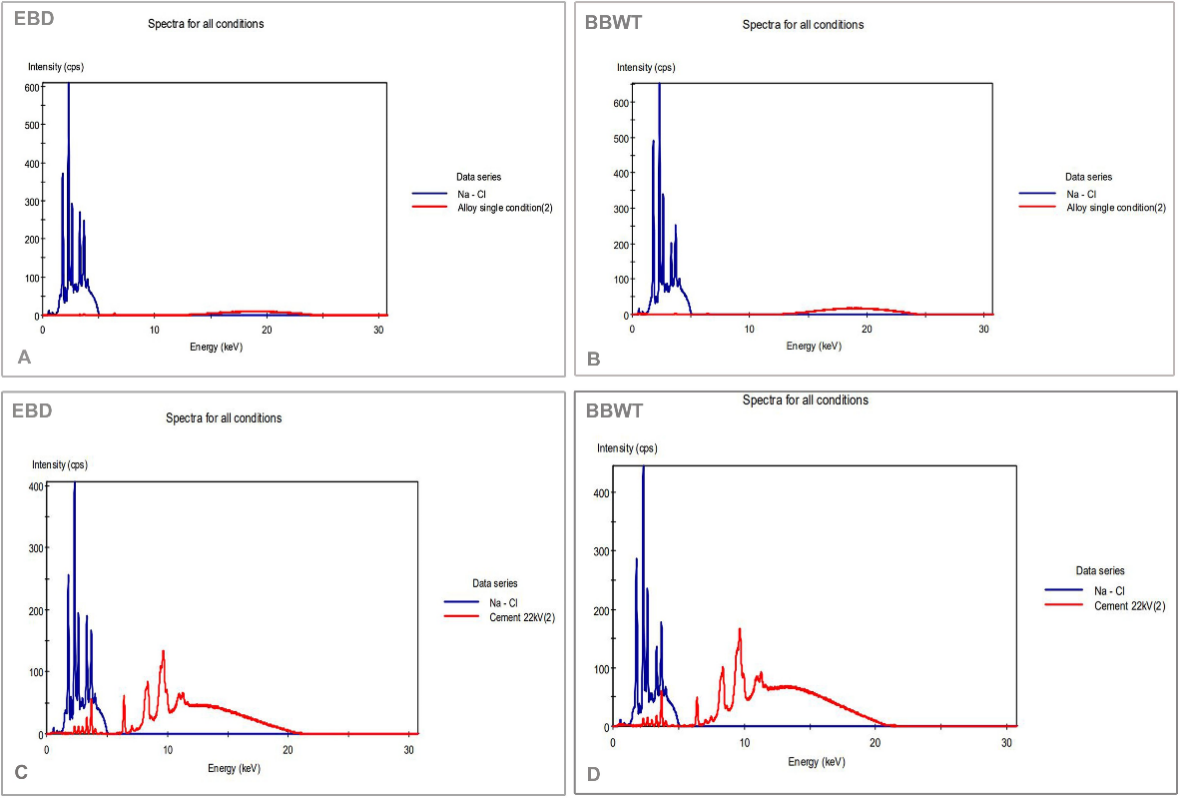
ED-XRF analysis of the elemental composition of the scaly shank skin (**A**, **B**) and of oxides concentration (**C**, **D**) in EBD and BBWT.

The elements [Al, Si, P, S, Ti, Cr, Mn, Fe, Ni, Cu, Zn, Se, Nb, Zr, Ag, Sn, Sb] have been discovered from the skin samples of EBD and BBWT. Sb, S, Ti, Al, Fe, Ag and Ti, S, Sb, Si, Fe, Ag, Al were the highest concentrations from the samples of EBD and BBWT, respectively. The concentration of the elements is recorded in Table 1 and Fig. 21A, B. On the other hand, the oxides concentration [Na2O, MgO, Al2O3, SiO2, P2O5, SO3, ClO2, K2O, CaO, TiO2, Cr2O3, Mn2O3, Fe2O3, ZnO, and SrO] in Table 2 and Fig. 21C, D showed that Mn2O3 was the dominant oxide, followed by SrO, and Cr2O3 in the skin sample from EBD. Moreover, the most appreciably high oxide in BBWT was Mn2O3, followed by Cr2O3, and SrO.

## Discussion

Just as the feathers are the principal integumentary structure of the avian species for their locomotion and protection against heat, mechanical, chemical, and biological effects in the air, the scaly featherless feet are fundamental for their movement and protection on the ground, water, and branches. The scaly feet are relevant for an adaptation for locomotion in foot-propelled aquatic birds^39^, movement during swimming^40^, and taking off and landing in flying birds^39^. Furthermore, scales of feet, feathers, and hairs regulate body temperature and offer a physical defense^41^.

The shank skin color was yellow in EBD and creamy-white in BBWT. It ranges from white, green, bluish, yellow, and grey to black in Korean native chickens, and is typically yellow in the European breeds^42^. The variations of the shank skin colouration are mainly due to a combination of certain genes that affect melanin and carotenoid pigmentation, unknown polygenic modifiers, and environmental influences (i.e., diet). The melanin pigmentation is influenced by melanocytes in the dermal and epidermal layers of the skin as supported in the studied shank skin of EBD, but carotenoid pigmentation is dependent on carotenoids in the diet^43,44^.

Our findings exhibited that the texture of the scaly shank skin of BBWT was harder than that of EBD, specifically at the dorsum and ankle regions, due to high keratinization of the scales and sulci. The difference in the shank skin texture between the studied birds indicates that the terrestrial birds are more subjected to mechanical influences than the aquatic birds. The avian scales should be sufficiently hard to provide mechanical protection but remain flexible enough to adapt to changes in the hydraulic pressure of the blood vessels and tissue in the foot and leg^45^. Generally, the scales on the avian shank are distinguished into 4 types; they differ in several aspects^23,46–48^. The same avian species have various scales on different areas of the plantar aspect^49^. The present study showed 2 types of scales in EBD: scute and scutella and 4 types in WBBT: scute, scutella, reticula, and cancella (interstitial). The variety of scales in WBBT represents an adaptation to its ecosystem. The heavy-scaled tarsometatarsi are more suited to the terrestrial lifestyle to provide greater flexible and tough surfaces^50^. The large scales in the studied birds were rectangular to hexagonal in shape as are noticeable in some avian species^17,22,49^. In ostrich, the shape of the scales ranges from a small dome to a large hexagonal, similar to the snake’s skin^2^. On the other hand, the scales overlap in WBBT (on the dorsum), chickens^12^, and picids, kingfishers (on the planter surface)^13^, while those of aquatic birds; ducks and geese butt against each other^12^. The overlapped scales occurred because of the increase in the deepening of the sulci and the size of the scale during the development^10^. Undoubtedly, the density of the scales on the leg differs depending on the species and habitats (more intense in terrestrial vs. aquatic) as documented in the two studied birds. The density of the scales is also extremely variable on the foot pads depending on the species, season, bird habitats, and the type of foraging habitat and behavior^49,51–53^.

Avian skin is firmly attached to the underlying structures on the extremities, beak, and feet, whilst over the remainder of the body, it is loosely located^10^. Histologically, the scaly skin of the two studied birds was somewhat similar to that described in the feathered skin of other avian species with some variations in thickness^10–13,54^. Generally, the stratum corneum of the epidermis is relatively thick in featherless body regions, corresponding with the mechanical forces to which these parts of the body are subjected^12^. Therefore, the stratum corneum of the dorsal and plantar skin aspects in EBD and BBWT was thicker than that of the other skin aspects. This layer (represented the scales) arises from the rapid proliferation of the underlaying stratum germinativum, which gives rise to closely-packed layers of keratinized cells as reported by Hodges^10^.

It is worth mentioning that, the dermal collagen fibers were denser and thicker in BBWT to suit the friction and loading forces they encountered, because collagen fibers endow strength and flexibility to the skin^55^ and mechanical strength to the dermis^12^. In contrast, the dermal adipose tissue was more plentiful in the shank skin of EBD, appearing in both layers of the stratum profundum to provide energy during swimming. As adipose tissue is an essential energy supply^56^. This tissue is not only a reservoir of energy but also plays an endocrine role in controlling hunger and energy expenditure among other bodily activities. Furthermore, it controls complex processes such as the immune response, inflammation, and reproduction^57,58^.

Although the dermal papillae are absent in the avian skin^11,54,59^, our results explained the appearance of distinct dermal papillae in EBD and a few delicate dermal papillae in BBWT. They are also observed in the feathered skin of the neck and back of ostrich^2^. However, König, et al.^12^ reported that discrete dermal papillae are found only in conjunction with feather follicles. The dermal papillae have blood vessels that provide nutrients to the epidermis and help control the skin’s temperature. Overall, the shank skin (specifically the ventral aspect) was highly vascularized in BBWT vs EBD due to the difference between terrestrial and aquatic ecosystems in temperature regulation. Thus, skin plays a pivotal role in thermoregulation^13,54,60^. The birds can lose heat through the skin (particularly on the legs)^12^.

Langerhans cells were observed within the stratum germinativum and dermis of BBWT. These cells are tissue-resident macrophages of the skin^61^, serve as the outermost guard of the skin’s immune system and induce initial reactions against pathogens that invade the skin^62^. So, the skin of terrestrial birds exhibited a high immune resistance against diseases and toxic agents. Moreover, abundant telocytes (TCs) were identified within the dermis of both birds closely related to fibroblasts, blood vessels, and connective fiber bundles. In accordance with the reports of Rusu, et al.^63^, and Manole and Simionescu^64^, these cells play pivotal roles in skin homeostasis^64,65^, blood vessel homeostasis^66^, angiogenesis^64^, renewal processes after skin injuries^67^, and protection against inflammation^68^.

Many previously published studies have clarified the elemental analysis of bird feathers^69–75^, but the data on the elemental analysis of avian skin are rare. Furthermore, few studies exist on the elemental content of mammalian skin. Even the elemental analysis across human skin is performed via electron probe analysis and analytical electron microscopy^76^. So, we discussed a novel method for assessing the avian shank skin of aquatic and terrestrial birds via ED-XRF procedure. Our results exhibited that elements and oxides were present in both species, with no discernible difference in some concentrations from the analyzed samples of EBD and BBWT. The concentration of the biological elements across the skin is influenced by habitats^77^ and several biological factors such as age and sex^76,78^. These elements have several proposed functions. For instance, sulfur contributes to skin keratinization^79,80^, sodium promotes epidermal wound healing^81,82^, and some elements have a role in skin conditioning. Some elements (Si, S, Fe, and Al) were recorded the highest concentrations in the shank skin of two studied birds. These elements are the main building blocks for resilience and protection against environmental friction. The current study also recorded the oxides concentration in the shank skin. The oxides have a significant role in the skin’s unique adaptability and function^83^.

The elemental analysis of the shank skin provides new insights into the potential use of the shank skin based on the previous literature of many authors^84–87^, who mentioned that dead poultry, feathers, eggshells, hatchery waste, and mechanically deboned residue may be converted into feedstuffs that are used in the poultry and pet food industries as animal protein source. The elemental composition of the scaly skin of the studied birds may be useful for mineral supplementation in the food industry. The mineral supplementation is crucial for preserving the optimal health of poultry and animals^88^.

## Conclusion

The morphological study of scaly shank skin has received less research attention and there are not enough data on this topic. To our knowledge, this work is the first to shed light on the comprehensive morphological study of the scaly shank skin of different bird habitats. The shanks of terrestrial birds are more tolerant to surrounding biological, mechanical, and chemical factors. ED-XRF analysis revealed that the shank skin is a good source of mineral supplementation in the food industry, whereas mineral supplementation is crucial for preserving the optimal health of poultry and animals. Furthermore, the shank skin could represent a promising source for extraction collagen and gelatin, to be used in the biomedical and pharmaceutical industries.

## Data Availability

Data are available on request from the corresponding author.
